# Spatial patterns of childhood obesity clusters linked to socioeconomic inequalities

**DOI:** 10.3389/fpubh.2025.1497090

**Published:** 2025-08-19

**Authors:** Orit Mazza, Chemda Gluck, Amir Haim, Robyn Jacob Bornstein

**Affiliations:** ^1^Loewenstein Rehabilitation Medical Center, Raanana, Israel; ^2^Clalit Health Services, Dan Petah-Tiqwa District, Israel; ^3^Gray Faculty of Medicine & Health Sciences, Tel Aviv University, Tel Aviv, Israel

**Keywords:** obesity, overweight, childhood obesity, geographical information systems, spatial analysis

## Abstract

**Introduction:**

The childhood obesity epidemic continues to be a challenge worldwide despite advances in prevention and treatment. Multifactorial causes are responsible for this epidemiology, and unequivocally, environmental factors play a key role. Studying the connection between socioeconomic factors and prevalence of childhood obesity is key to instituting change on a public health level.

**Objective:**

To identify geographical areas (clusters) with high and low prevalence of childhood obesity and examine their spatial association with socio-economic and demographic factors.

**Methods:**

Cluster analysis of geographic and population data for localities and regional councils was performed using growth data for children in grade 1. Analysis of childhood obesity prevalence utilized spatial autocorrelation (Moran’s I) and Getis-Ord $G_{i}^{*}\;$ statistic (hotspot analysis). Local Geographically Weighted Regression (GWR) and Multiscale GWR (MGWR) were performed to examine socio-economic and demographic predictors of the z-score values from the hotspot analysis.

**Results:**

The cluster analysis identified several significant spatial clusters of localities with high and low z-scores from the Getis-Ord $G_{i}^{*}\;$ hotspot analysis. Both the GWR and MGWR models demonstrated notable spatial variation, achieving high adjusted R-squared values (95.2 and 96.4%, respectively) and low residual variances (0.05 and 0.03, respectively). The analysis indicated that the variables exhibited a significant localized effect (*p* < 0.05), underscoring spatial heterogeneity. Among these variables, three showed a significant influence across the entire geographic area: average years of education for individuals aged 25–54, the percentage of families with four or more children, and the socioeconomic index. These findings emphasize the spatial variability of these factors and the ability of the model to generate a range of coefficients tailored to each locality.

**Conclusion:**

The application of geographic techniques enables examination of spatial patterns of childhood obesity. The current analysis is the first study that demonstrate a direct association between cluster areas of childhood obesity prevalence and socio-economic and demographic factors in the Middle East. It highlights that spatial dependence and heterogeneity are key factors in analyzing patterns of childhood obesity. Correlations between sociodemographic parameters are consistent with patterns observed in high-income countries (a negative socioeconomic index association) and in middle-and low-income countries (a positive association with average years of schooling). The results suggest that the immediate geographic environment plays a substantial role in childhood obesity. Therefore, it may reflect different patterns related to macroeconomic factors, such as the country’s income level.

## Introduction

Childhood obesity is becoming an increasingly alarming global health concern. Over recent decades, the worldwide prevalence of obesity has risen dramatically. This growing epidemic contributes significantly to both morbidity and mortality, placing a heavy burden on healthcare systems and economic resources (1, 2). According to the World Health Organization (WHO), only 4% of children and adolescents aged 5 to 19 were classified as overweight or obese in 1975. By 2016, that number had risen sharply to over 18%. This rise is linked to the earlier onset of related health conditions, increasing the risk of chronic diseases and premature death in adulthood (3). Projections based on data from 191 countries estimate that by 2030, up to 30% of children globally could be overweight or obese. The situation is more concerning in middle and high-income countries, where prevalence could reach as high as 58% (4).

Global economic development and modernization have led to widespread changes in socioeconomic environments, contributing to widespread lifestyle changes that have resulted in an increase in childhood obesity over time and across regions (4). These trends are contributing to a significant global economic burden. It is estimated that the cost of overweight and obesity could rise from 2.19% to 3.3% of global GDP across 161 countries by 2060. However, stabilizing obesity rates at 2019 levels could generate annual global savings of approximately $2.2 trillion. This underscores the urgent need for strategic global investment and resource allocation to address this growing issue (5).

Obesity is a complex, chronic condition with multiple contributing factors and serious secondary health complications. It is associated with numerous non-communicable diseases (NCDs), including insulin resistance, diabetes, hypertension, cardiovascular disease, liver dysfunction, and various cancers. It also leads to musculoskeletal issues such as arthritis, postural changes, and lower limb pain (6–8). Among children who suffer from obesity, there is a significantly elevated likelihood of remaining obese into adulthood (9).

While weight gain results from an imbalance between calorie intake and expenditure, environmental and ecological factors also play a substantial role (10). Current interventions primarily focus on lifestyle changes that target obesity-related behaviors, such as improving dietary habits and increasing physical activity (11). However, this treatment paradigm varies widely in terms of implementation and efficacy. Despite these interventions, obesity prevalence continues to rise, requiring additional research and the development of novel strategies for both prevention and treatment (12, 13).

In the past decade, there has been a new focus on management strategies for obesity that utilize geographic data (12). Such research has revealed a new perspective on obesity, redefining disease management from an individual model to a public health strategy that seeks to modify the obesogenic environment (14). In 2022, the WHO also acknowledged the importance of social responsibility for obesity development and control in its framework for the prevention and management of obesity (1).

An obesogenic environment promotes obesity by increasing the likelihood of weight gain. It discourages physical activity, encourages a sedentary lifestyle and passive behaviors, and fosters habits that lead to a positive calorie balance, ultimately resulting in weight gain (15). Neighboring regions often share similar characteristics, and likely to be correlated, resulting in clustering effect. Identifying spatial clustering of childhood obesity can help identify priority areas for intervention and management at the community level (16). The neighbourhood environment is composed of several components that can impact health and create an obesogenic environment for children. Environmental factors can influence both diet and physical activity, which may contribute to weight gain. These include aspects of the built environment, such as access to parks, recreational areas, public transportation, subways, and bus stops. In addition, food environment factors like the availability of convenience stores, supermarkets, grocery stores, fast food outlets, and fruit and vegetable markets affect where and what people eat, whether at home, school, restaurants, or stores. Together, these factors shape daily habits and opportunities for healthy eating and physical activity (17, 18). Broader macro-level environmental factors, such as food marketing, agricultural policies, food production, food distribution, and socioeconomic factors, also influence these local factors (19).

Analyzing the spatial distribution of childhood obesity can identify environmental and contextual factors that influence choices regarding nutrition, healthy eating, and opportunities for physical activity. The modernization-globalization theory suggests that global changes in socioeconomic environments have contributed to these shifts in childhood obesity over time and across geographic regions, creating gaps in understanding how local factors and global obesity trends interact (4). This effect is especially true in the Middle East region (20).

Several studies have examined the interaction between geographical locations, childhood obesity prevalence, and socioeconomic factors. These studies show that geographic variation in childhood obesity is associated with socioeconomic disparities, exists both across different countries and within districts of the same country (10, 21–26). In industrized regions, studies have identified a negative relationship between socioeconomic status and childhood obesity (27, 28). However, the effect of spatial clustering on childhood obesity has not been thoroughly investigated, and the findings regarding childhood obesity in developed societies are inconclusive. For example, in England, variations in childhood obesity prevalence were observed across regions, and this was linked to socioeconomic factors such as population density, non-white ethnicity, and unemployment level (21). In China, similar findings showed that children from high socioeconomic status were more likely to be obese than those from moderate to low socioeconomic groups (23). In Thailand, childhood obesity clusters showed a notable and positive association with regions characterized by elevated levels of socioeconomic environmental factors, such as annual income, population density, number of households, education level, and unemployment rate (22).

These findings suggested that the relationship between childhood obesity and socioeconomic status is complex and varies depending on the location. While such studies highlight the value of analyzing geographic clustering and socioeconomic influences, and provide insights into how location affects childhood obesity, these studies did not examine this relationship in the Middle East.

Moreover, the association between obesity and socioeconomic factors differs based on gender, age, and country (28–30). In Middle Eastern countries, higher socioeconomic status is associated with higher childhood obesity rates (20). However, the evidence is inconsistent, and the spatial effect, considering the autocorrelation expected in geospatial analysis, was not taken into account (31). Spatial modelling is essential in this context because it accounts for spatial autocorrelation in geospatial data. When spatial dependence is ignored, researchers often rely on linear models that assume independent residuals. This assumption is usually violated in spatial analyses, resulting in inaccurate conclusions.

This study addresses the gap by focusing on childhood obesity and its relationship with socioeconomic factors at the neighbourhood level. Childhood obesity was defined by a body mass index (BMI) above the 97.7th percentile, based on WHO growth curves adjusted for age and gender (32). The study employs a research framework that utilizes geographical techniques to inform policymaking by developing a model designed to modify the obesogenic environment for children. A population-level index was used to support effective decision-making and policy implementation, particularly by central governments collaborating with local authorities. While this index helped synthesize diverse factors, it was not sufficient on its own. Therefore, a socioeconomic index and multiple sociodemographic and economic indicators were used to enhance interpretability (15–20).

The objective of the study was to identify areas with a high prevalence of obesity, as determined through thematic maps and analysis of geographical clusters of childhood obesity prevalence, which could serve as potential targets for intervention. These clusters were then analyzed in relation to socioeconomic status. Cluster detection helped map the spatial distribution of obesity prevalence, enabling subsequent analysis of socioeconomic-demographic data by region. This is the first study to apply geographical techniques in analyzing childhood obesity clusters in this region. Consequently, it sheds light on the patterns of childhood obesity clusters and aids in understanding how inequalities of regional socioeconomic status are associated with childhood obesity. Spatial regression modelling, which accounts for spatial autocorrelation was used, making it more suited for geographic data than traditional non-spatial regression methods (21).

## Methods

The study was approved by the Institutional Ethics Committee on Human Research at the Loewenstein Rehabilitation Medical Center (No.LOE-21-0012) with Ministry of Health regulations, the institutional ethics committee did not require written informed consent because the data was collected anonymously from digitalized medical files, in the absence of active patient participation.

### Study area and the study population

Israel is a developed nation with a population influenced by successive generations of immigration, resulting in a diverse and multi-ethnic population (33). Despite a universal healthcare model that has provided comprehensive health insurance to all Israeli citizens since 1995, profound health disparities persist across subpopulations. Differences include geographic maldistribution of health resources (for example, acute care hospital beds), and access to healthcare information and services (34). There are also disparities between the peripheral and central areas of the country, with significantly poorer health outcomes in the peripheries. Contributing factors include lack of medical knowledge, low access to health services, low consumption of health services, and higher rates of chronic illness (35).

This research utilized data on the childhood prevalence of obesity over a five-year period, from 2014 to 2018, across a broad geographical area within the country.

### Databases

The regular operations of the Student Health Service in Israel primarily focus on evaluating and measuring students’ growth. Public health nurses conduct growth assessments for students in both the first and seventh grades (36). From 2014 to 2018, the Ministry of Health in Israel managed a Business Intelligence (BI) Data system that encompassed data from these assessments, including the following parameters: the year of interest, age group (first or seventh grade), and geographic location (37).

It is essential to highlight that the most recent data available was used in this study. The database supports research by allowing the examination of trends over time (2014–2018) and providing access to geographical information related to childhood obesity (37). The raw data extracted from this system (retrieved on January 1, 2022) can be found in the following Excel spreadsheet, which has been translated into English: https://github.com/OritMazza/childhood_obesity_Israel. Data were displayed as obesity percentages for each locality among students in grades one and seven, across 5 years from 2014 to 2018. Authors had no access to information that could identify individual participants during or after data collection. From this initial dataset, a subset for analysis was derived, containing the data points for grade 1 students. The choice to focus on the 6–7-year age cross-section (first grade) stems from the rationale that early intervention is known to yield better outcomes. Changes should be instituted as early as possible to establish healthy habits, prevent obesity complications, reduce stigma, and involve parents and the educational environment early (38, 39).

### Geographic data

The localities layer (last updated as of May 2018), which contains geographical units based on the jurisdictional boundaries of local authorities such as municipalities, local councils, and regional councils, was downloaded from the government website of the Ministry of Interior at the following link: https://www.gov.il/he/departments/guides/info-gis.

Each regional council governs multiple settlements scattered across rural areas. Since obesity data were provided to regional councils rather than individual settlements, all settlements within each regional council were consolidated into a single polygon.

Missing localities were identified in the previously mentioned layer sourced from the Ministry of Interior’s website. The missing data were in the statistical areas layer from the Central Bureau of Statistics (40). To better align with geographical boundaries rather than statistical units, all statistical units was consolidated for each locality into a single polygon. The localities within these polygons, sourced from the census, include: Oranit, Alfei Menashe, Elkana, Efrat, Ariel, Beit El, Beit Aryeh, Beitar Ilit, Givat Ze’ev, Har Adar, Ma’ale Adumim, Ghajar, Emanuel, Kiryat Arba, and Kedumim. Geographical areas lacking published information on childhood obesity prevalence are primarily comprised of industrial zones, areas under construction, or vacant spaces.

The localities for which specific, locality-level data on obesity prevalence have not been published include: Migdal Tefen, Metula, Mi’ilya, Eyn Mahel, Eyn Kinya, Rajar, Ramat Negev, Elyahin, Harish, Yesud Hama’ala, Kefar Shmaryahu, Tamar, Binyamina—Giv’at Ada, Neot Hovav, and Rosh Pina.

### Incremental spatial autocorrelation

Spatial dependence within elements in geographic regions is a fundamental concept in Geographic Information Systems (GIS). Spatial dependence, namely spatial autocorrelation among attributed geographic objects, is essential for examining whether spatial pattern relationships exist or reflect random spatial patterns, which can lead to false-positive cluster dependencies and misinterpretation. This first step is applied before spatial analysis examines the local spatial associations to detect spatial heterogeneity between locations (41–43). To determine the optimal critical distance for the local cluster investigation, a spatial autocorrelation test was conducted using Moran’s index (44). This method was used to determine optimal spatial autocorrelation before spatial local clusters of high or low values for childhood obesity analysis are identified using Getis-Ord $G_{i}^{*}$ (45).

Spatial autocorrelation tests depend on the spatial weight matrix that is chosen. It defines the geographical distance chosen for autocorrelation patterns. Therefore, Moran’s I can be calculated for different distance bands, resulting in different spatial dependencies (43). To overcome this gap, global Moran’s I was tested for a serial array of distances (incremental autocorrelation) to find the most optimal spatial pattern in which the geoelements resemble the highest dependency in space (represented by z-score and *p*-value). This value was used for the fixed distance band, which was used later in the cluster analysis using Getis-Ord $G_{i}^{*}$ .

Geary’s C is another global measure of spatial autocorrelation. However, unlike Moran’s I, it has not been widely adopted in public health studies (43). Moreover, when the focus is on identifying spatial clustering and detecting strong positive spatial patterns, Moran’s I has been shown to outperform Geary’s C (46). Getis-Ord G is based on equivalent formulae for analytical distributions, as for Moran’s I and Geary’s C. However, it is considered a more complicated approach to analyze spatial autocorrelation (43).

Distance sensitivity analysis was done in two steps to find to most meaningful spatial scale.

First, distance intervals of 10,000 m were tested, and the z-scores of each distance were calculated. Z-score indicates the intensity of spatial clustering for a given distance threshold for spatial weights. A total of 30 distance intervals (the maximum number of intervals that can be examined through the software) were calculated, ranging from 1,000 m to 300,000 m. This range of distance enables to examine the distance band when considering a vast geographic area that encompasses the entire country. During the second stage, a more accurate distance band was examined by 1,000-meter intervals from 40,000 m to 70,000 m (30 intervals). The Optimal distance was subsequently used to define the fixed distance band used for Getis & Ord $G_{i}^{*}$ equation. More specifically, the graph was used to identify maximum points where the z-score rises and then descends, which helped in selecting the threshold distance for defining neighbors between geographic areas. Moran’s I test was examined for 2014 and the same threshold was used to test the spatial relationship of childhood obesity in 2018, enabling a comparison of the geographical distribution of childhood obesity between those years. The global autocorrelation, as measured by Moran’s I, addresses the question of spatial dependence. However, to examine how spatial correlation varies from one study area to another, a local analysis is required to detect hot and cold spots (45).

### Spatial analysis

The prevalence of childhood obesity by geographic area was examined using Geographic Information Systems (Esri Inc., ArcGIS pro 2.8.0 desktop). Cluster analysis was performed according to the Getis-Ord $\;G_{i}^{*}$ equation by spatial connections defined by the fixed-distance band method. In this method, each pair of observations was assigned a binary value of either 0 or 1 based on whether the distance between the observations was determined to be critical to the spatial unit (geographic area). If the variable for a given geographic region was defined as outside the critical distance, it received a value of 0 with no subsequent effect on the calculation. If the variable was defined as below the threshold, it received a value of 1 and was included in the final statistical calculation. A fixed distance band may be suboptimal when geographic units within the study area vary in size (47). Despite variations in geographic unit sizes, there is a reasonable degree of spatial homogeneity in our study area concerning the z-score of childhood obesity prevalence and sociodemographic factors. The assumption is that environmental and demographic factors create a relatively similar region that creates clusters of similar areas. Therefore, a fixed distance band was used to capture local spatial relationships effectively. Additionally, a fixed distance band simplified the computational process, and the interpretation of results compared to variable bandwidth methods.

A sensitivity analysis was conducted to evaluate how different distance bands affect the results of cluster analysis and subsequent regression analysis for 2014. The distance bands used in this test were selected based on the peaks identified in the Moran’s Index analysis. Cluster analysis was performed on localities by prevalence of childhood obesity. For each object (locality), hotspot analysis was performed by the Getis-Ord $G_{i}^{*}$ statistic, Equation 1 (48, 49). The Getis-Ord $G_{i}^{*}$ method identifies clustering patterns by highlighting hot spots and cold spots, in contrast to the Local Moran’s I method (LISA), which also detects Low-High and High-Low outliers (50). Since this study focuses specifically on detecting clusters of high childhood obesity (i.e., High-High or Low-Low interactions), the Getis-Ord $G_{i}^{*}$ method is more appropriate for our objectives. The resulting z-scores and *p*-values were used to identify spatial clusters of both high and low prevalence of childhood obesity. The Getis-Ord $G_{i}^{*}$ statistic was chosen as the method for cluster identification in order to identify localized clusters of high or low values of childhood obesity in the data. In addition, this approach will help set future policies in childhood obesity management at the population level and set priorities.

Equation 1 [Getis & Ord $G_{i}^{*}$ local statistic]


(1) $$G_{i}^{*}=\frac{\sum_{j=1}^{n}w_{i,j}x_{j-}\bar{x}\sum_{j=1}^{n}w_{i,j}}{S\sqrt{\frac{n\sum_{j=1}^{n}w_{i,j}^{2}-{\left(\sum_{j=1}^{n}w_{i,j}\right)}^{2}}{n-1}}}$$

$$\bar{x}=\frac{\sum_{j=1}^{n}x_{j}}{n}$$

$$S=\sqrt{\frac{\sum_{j=1}^{n}x_{j}^{2}}{n}-{\left(\bar{x}\right)}^{2}}$$

In the Getis-Ord $G_{i}^{*}$ equation, *n* is equal to the number of objects being examined (localities). i and j are indices of the geographic localities. x *_j_* specifies the variable value of the percentage (prevalence) of childhood obesity in each locality j. *w_i,j_* is the spatial weight matrix of the geographic areas i to j. The value of $G_{i}^{*}$ represents the resulting z-score.

A hotspot was defined as a locality with a high prevalence of childhood obesity, which is also surrounded by other localities with high childhood obesity prevalence. A coldspot was defined as a locality with a low prevalence of childhood obesity, surrounded by other localities with a low prevalence of childhood obesity. A positive z-score indicated a deviation from the average in terms of standard deviations and suggested a higher prevalence of obesity within the specified spatial window for cluster investigation, as opposed to a single location. False discovery rate correction was applied to account for multiple testing and spatial dependence. The FDR correction estimated the number of false positives for a given confidence level and adjusted the critical *p*-value. Features are displayed with statistical significance levels of 99, 95, and 90% confidence. The clustering for features that do not meet these thresholds is considered not statistically significant. Significance of local clustering was based on a *p*-value < 0.01 for the 99% confidence level.

### Thematic (choropleth) maps

Geographic maps were compared presenting the childhood obesity prevalence by locality in 2014 and 2018 in Israel, using five classes based on quantile breaks. The quantile breaks method distributes the observations equally across the class interval, giving unequal class widths, however the same frequency of observations per class. This method is highly suited for choropleth map-reading tasks for classifying epidemiological data (51).

The results of the hot spot analysis and the regression analysis, including predicted values, coefficient residuals, and regression coefficients, were presented as thematic maps. These maps highlighted clusters of localities with similar obesity prevalence, whether high or low, for the years 2014 and 2018. The maps for these specific years were selected to observe temporal trends and uncover spatial patterns related to childhood obesity.

### Statistical analysis

The Shapiro–Wilk test, performed using the *shapiro_test()* function, was used to evaluate the normality assumption for each year. To examine differences in median obesity prevalence across localities from 2014 to 2018, the Kruskal-Wallis test was conducted using the *kruskal.test()* function. Statistical analysis was done with the R package *rstatix* (52). In addition, the R packages *tidyverse* and *ggpubr* were used for data visualization (53, 54). A *p*-value < 0.05 level was considered statistically significant. Correlations among the independent variables that were used later in the regression analysis, were tested by the *cor()* function within the R package *caret* (55). Variables with a high correlation (r > 0.7) were excluded from the analysis, while the remaining variables were included in the regression analysis. The highly correlated variables were found with the *findCorreltion()* function within the R package *caret.*

The source codes for the analyses can be found at: https://github.com/OritMazza/childhood_obesity_Israel.

### Regression analysis

In order to analyze the relationship between the clustering effect of childhood obesity and socioeconomic factors of interest, the socioeconomic factors of the localities, were combined with the data from the hotspot analysis (z-scores) from 2014. The data was combined according to the names of the localities. There was no missing data. The socioeconomic factors were derived from the Israel Central Bureau of Statistics publication that presented findings of a study on characterization and ranking of various geographical units in Israel by the population’s socio-economic level in 2015 (56). It included 14 demographic (median age, dependency ratio, percent of families with 4 children or more), social (average years of schooling of persons aged 25–54, percent of academic degree holders of person aged 25–54), economic parameters (percent of wage and income earners of persons aged 15 and over, percent of women aged 25–54 with no income from work, percent of wage and income earners above twice the average wage, percent of subminimum wage earners, percent of recipients of income support and income supplement to old age pension), and other general parameters (average monthly income *per capita*, rate of motorization, average vehicle licence fee, and average numbers of days aboard). Those variables were standardized, and the z-score of each variable was analyzed. The socioeconomic index was scored on an ordinal scale from 1 to 10, from low to high socioeconomic status, respectively.

The method of selecting socioeconomic variables for obesity research analysis is both controversial and lacks uniformity (57). Utilizing a socioeconomic index that integrates multiple variables, including social demographics, can enhance interpretation and facilitate the implementation of diverse policies by the central government, specifically concerning local authorities. This approach, employing a similar index, is adopted by various countries worldwide for policymaking (e.g., Australia, New Zealand, Great Britain) (58). In this research paradigm, which involved applying geographical techniques to aid policymaking by presenting a model for a public health strategy aimed at altering the obesogenic environment for children. The use of a comprehensive index related to population level, rather than individual persons, is deemed suitable for contributing to population-level management, in our opinion. Further detailed explanation of the calculated index can be found at publication No. 1765 of the Israel Central Bureau of Statistics (56).

However, a single index is insufficient to investigate the influence of socioeconomic and demographic factors on childhood obesity, and limited information exists on the effect of income due to the scarcity of geographical studies exploring this association using geographical techniques (15–20). Therefore, all 14 demographic were included, social, and economic parameters in our analysis. To enhance the reliability of our findings, correlation tests were conducted to reduce multicollinearity and strengthen our conclusions.

The z-score for 2014 was calculated from the hotspot analysis to identify geographic clusters with high or low obesity prevalence. The results of the Getis-Ord $G_{i}^{*}$ analysis was combined with the socioeconomic and demographic parameters for the regression analysis according to the locality name and merged to a table that further deployed at the ArcGIS software for the regression modelling. The GWR was used to model spatially variable relationships that provided a local model of the variables by fitting a regression equation to every feature in the dataset (59).

The study used GWR to evaluate spatial relationships between neighborhoods and understand local variations in influencing factors. The model’s performance was assessed using several diagnostic metrics: Adjusted R-squared (AdjR^2^) measured the model’s goodness of fit, Akaike Information Criterion (AIC) identified the best-fitting model, with lower values indicating better fit, Sigma-Squared and Sigma-Squared Maximum Likelihood Estimate (MLE) estimated the residual variance, with lower values preferred for accuracy and Pseudo-t statistics tested the statistical significance of coefficients at a 95% confidence level. The diagnostics provided a comprehensive evaluation of the GWR model’s fit and accuracy. Additionally, the MGWR model was used for further analysis. MGWR extends GWR by allowing explanatory variable coefficients to vary across space and at different spatial scales. By assigning distinct bandwidths to each variable, MGWR revealed spatially unique influences, offering deeper insights into local variations in regression coefficients.

## Results

Two hundred and forty two localities were included in the analysis. The mean socioeconomic index was 4.9 ± 2.4.

All other variables that included in the final analysis were standardized. Accordingly, the median was 0 and the standard deviation was between 0.8 to 1. The first Moran’s I test, conducted with 10,000 m intervals up to a range of 300,000 m, displayed its highest peak at a distance band of 51,000 m. The results of this analysis are illustrated in Supplementary Figure S5. The second Moran I test (distance range from 40,000 m to 70,000 m with 1,000 meters intervals) exhibited a more sensitive distance band for a smaller zones of localities clusters.

**Figure 1:**
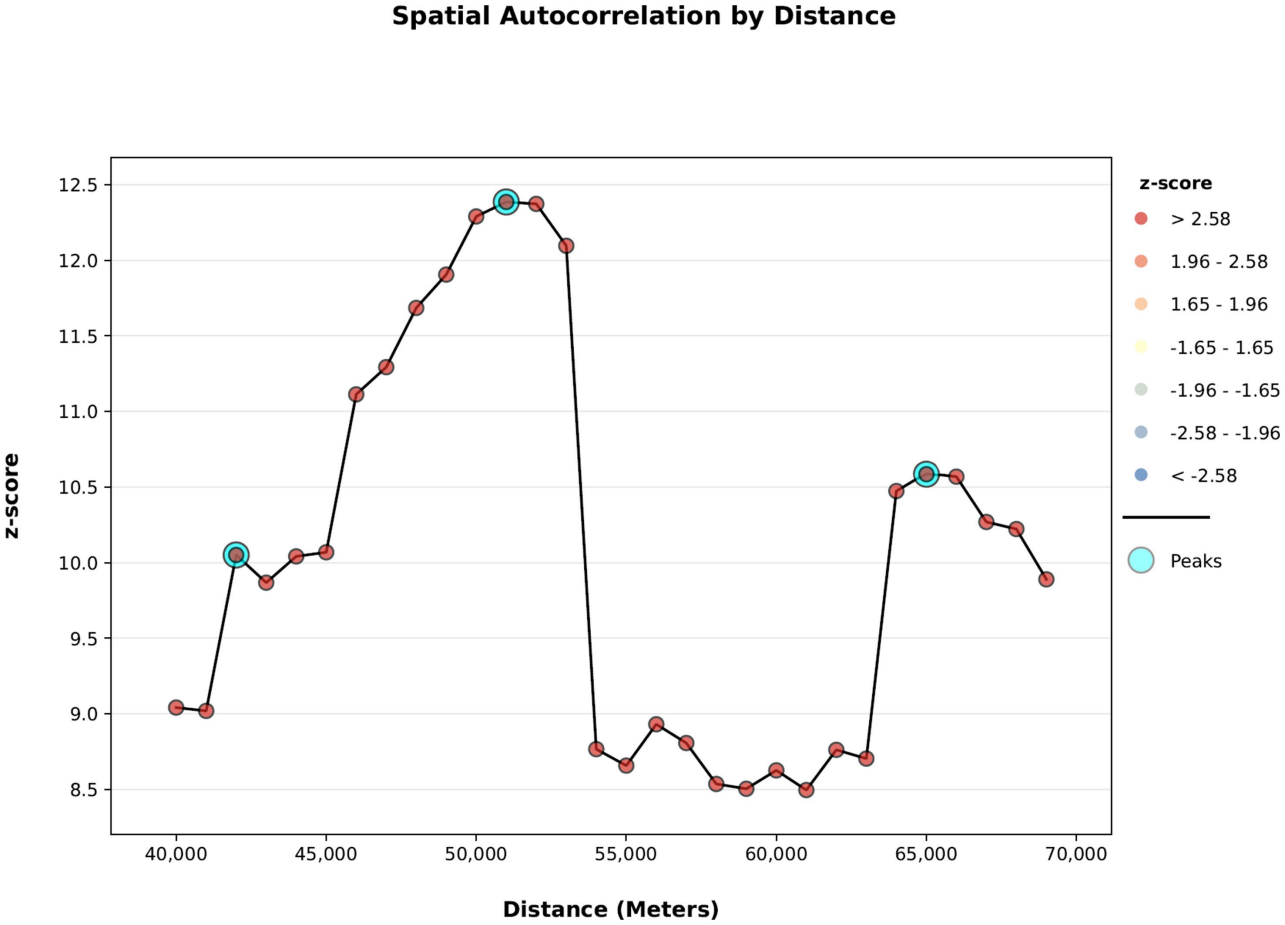
Z-score as a function of distance according to the spatial autocorrelation test (Moran’s I) for 2014 with a distance range between 40,000 m to 70,000 m.

Figure 1 shows a z-score graph as a function of distance according to Moran’s index, results of a spatial autocorrelation test for 2014 with a distance range between 40,000 m to 70,000 m. Each peak represents a potential spatial cluster. Three peaks were found: the first at 42,000 m, the second at 51,000 m, and the third at 65,000 m. The second peak reflected a broad trend, such as differences between north and south, but also was sensitive to nearby communities. Therefore, this peak was chosen as the critical distance in this study. Furthermore, the second peak corresponded to the highest value in the Moran’s I graph at this figure, indicating the greatest variation among geographic clusters. Therefore, it was specified as the critical distance band between neighbourhoods in the cluster analysis using the Getis-Ord $G_{i}^{*}$ Equation 1 in the hotspot analysis of childhood obesity in Israel. This critical distance was also chosen for the hotspot analysis of 2018 to enable comparison with the 2014 results.

**Figure 2:**
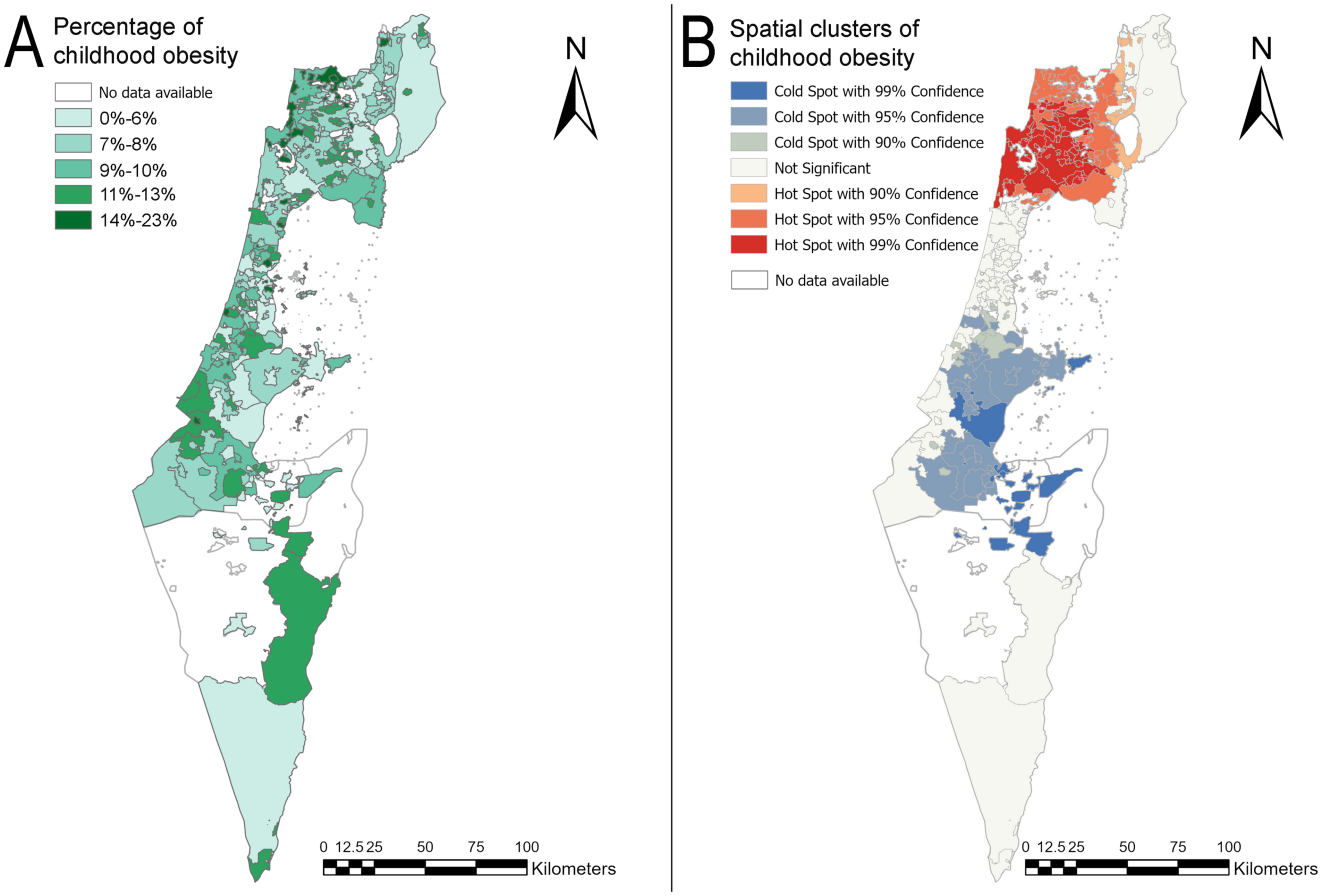
Childhood obesity prevalence map and hotspot analysis map by locality for the year 2014. **(A)** Prevalence map of childhood obesity according to the localities in 2014. **(B)** A hotspot analysis map of localities with childhood obesity in 2014. The basemaps reprinted from https://www.cbs.gov.il/EN/Pages/default.aspx under a CC BY license, with permission from the Central Bureau of Statistics (CBS) and the Ministry of Interior, State of Israel, original copyright (1997–2024).

Figure 2A describes childhood obesity percentages by locality for the year 2014 using 5 classes according to quantile breaks method. Figure 2B demonstrates a hotspot analysis map for localities according to obesity prevalence for 2014. Geographic hotspots with statistically significant obesity prevalence are marked in red, while geographic coldspots areas, where obesity prevalence is statistically significantly lower, are marked in blue. A hotspot cluster was identified in the northern of Israel, and additional coldspot clusters were detected at the center of Israel. There were 55 hotspot localities and 14 coldspot localities in 2014. A full list of the hotspot’s localities and coldspots localities for 2014 is shown in Supplementary Table S1.

**Figure 3:**
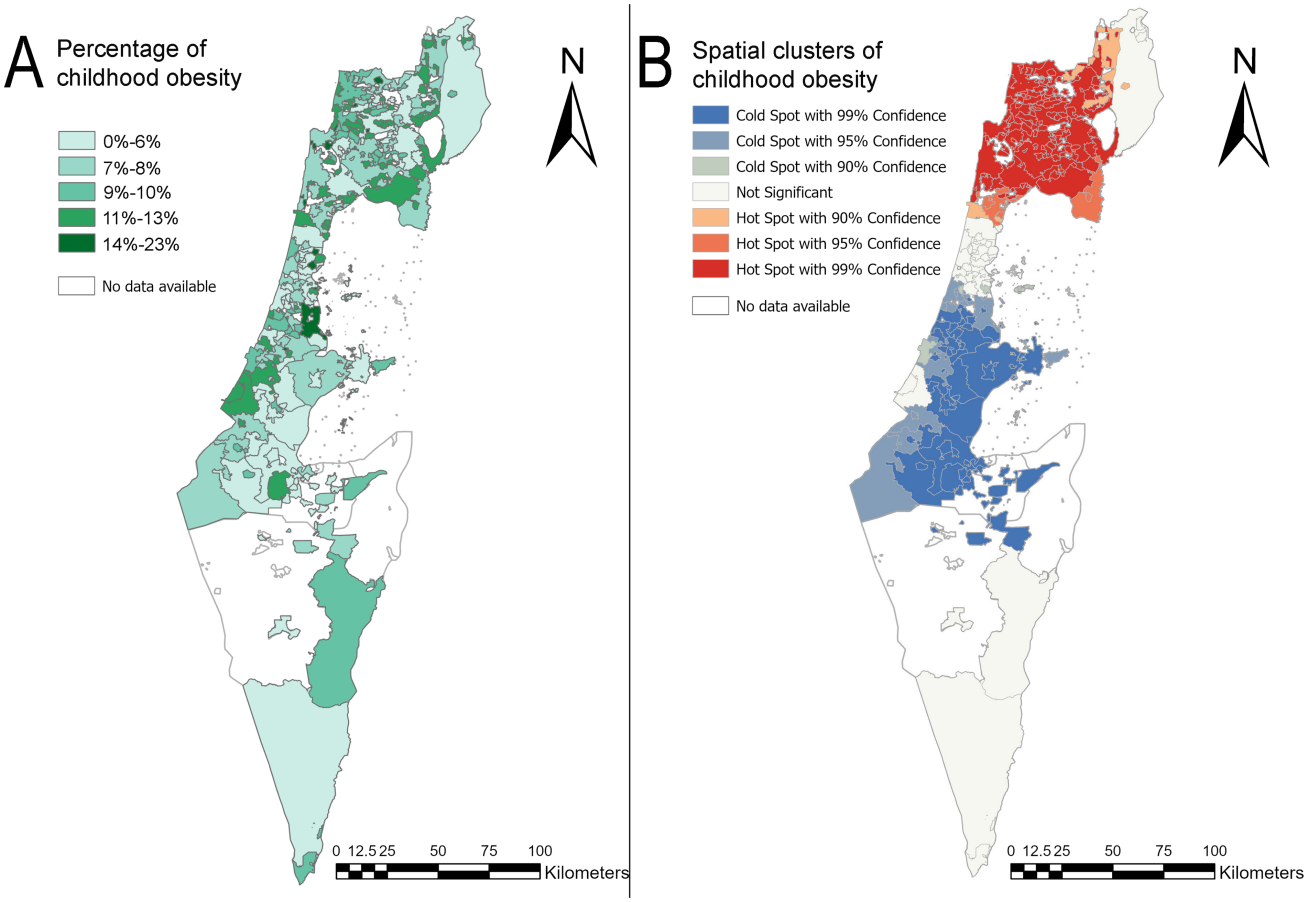
Obesity prevalence map in children and hotspot analysis map by locality for the year 2018 in Israel. **(A)** Map of childhood obesity prevalence by locality in 2018. **(B)** A hotspot analysis map of childhood obesity by locality in 2018. The basemaps reprinted from https://www.cbs.gov.il/EN/Pages/default.aspx under a CC BY license, with permission from the Central Bureau of Statistics (CBS) and Ministry of Interior, State of Israel, original copyright (1997–2024).

Figures 3A,B illustrate the map of childhood obesity prevalence by locality and the map of the hotspot analysis for 2018. These figures demonstrated changes in both regions of the country: the north and the center in comparison to the 2014 maps. In both regions, the sizes of the clusters were increased. In the case of geographic hotspots, more localities composed the cluster. Specifically, more localities with high values of childhood obesity prevalence were adjacent to other localities with similarly high values of obesity prevalence. Similarly, in the center of the country, a significant increase in the size of the geographic coldspots area has been observed, extending from the Hevel Modi’in region to the Eshkol Regional Council area. There were 97 hotspot localities and 59 coldspot localities in 2018. A full list of the geographic hotspots and coldspots localities for 2018 is shown in Supplementary Table S2. The Shapiro–Wilk test for normality showed statistically significant results (*p* < 0.002), indicating that the assumption of normality was violated. As a result, the Kruskal-Wallis test was conducted, which revealed no significant results [χ^2^(df = 4) = 8.87, *p* = 0.06]. Table 1 shows the average obesity percentage for each year and the standard deviation of the averages.

**Table 1:** Average obesity percentages in localities in Israel for the years 2014–2018.

Year	Average (%)	Standard deviation (%)
2014	0.09	0.041
2015	0.089	0.039
2016	0.083	0.037
2017	0.084	0.041
2018	0.083	0.038

After creating a correlation matrix as described in Supplementary Table S3. The eight highly correlated variables (r > 0.7) were excluded from the analysis (average monthly income *per capita*, percent of wage and income earners above twice the average wage, percent of academic degree holders of person aged 25–54, average numbers of days aboard, percent of subminimum wage earners, rate of motorization, percent of wage and income earners of persons aged 15 and over, percent of women aged 25–54 with no income from work). Therefore, the following variables were used in the spatial regression analysis: median age, dependency ratio, percent of families with 4 children or more, average years of education of persons aged 25–54, percent of recipients of income support and income supplement to old age pension, average vehicle licence fee, and socioeconomic index.

The results of both the MGWR and GWR models are summarized in Table 2. The lower AICc of the MGWR(−38.2) model suggests it captures spatial variation more effectively than the standard GWR (32.4). Both models show high R-squared values, with R-squared at 0.97 and adjusted R-squared at 0.95 for GWR and 0.96 for MGWR, indicating that the MGWR model explains a slightly greater portion of the variance in the data. This result highlights that incorporating multiscale spatial variation contributes significantly to explaining previously unexplained variation. Furthermore, the MGWR model has a lower Sigma-Squared (0.03) compared to GWR (0.05), indicating lower residual variance, though both models share the same Sigma-Squared MLE. The MGWR model’s higher effective degrees of freedom (EDF), at 181.84 compared to 155.02 for GWR, suggests that it allows for more localized variation. However, it is important to note that higher EDF alone does not necessarily imply better performance, as it could also indicate overfitting or unnecessary complexity. In this case, the lower AICc value for MGWR supports the conclusion that the additional flexibility introduced by the higher EDF represents meaningful spatial heterogeneity rather than overfitting.

**Table 2:** Statistical results of spatial regression models.

Statistic	GWR	MGWR
R-squared	0.97	0.97
Adjusted R-squared	0.95	0.96
AICc	32.38	−38.19
Sigma-squared	0.05	0.03
Sigma-squared MLE	0.03	0.03
Effective degrees of freedom	155.02	181.84

Both models effectively predict the z-score of childhood obesity prevalence, with MGWR showing a slight advantage over GWR. Based on this, the regression model’s ability to predict the outcome variable was tested by integrating the predictive variables using the GWR model. The model was then fine-tuned and further examined using MGWR. The GWR model assumes a constant spatial scale, applying the same bandwidth across the analysis. This consistency enables the creation of a prediction map that integrates the entire model. In contrast, MGWR allows different spatial scales for each variable, facilitating deeper exploration of the relationships between the regression variables and the outcome variable (60). MGWR also enables the creation of coefficient maps for each variable, allowing for a more detailed analysis and understanding of their effects. As a result, MGWR provides slightly better statistical accuracy than the GWR model, along with enhanced interpretability of the variable-specific impacts.

**Figure 4:**
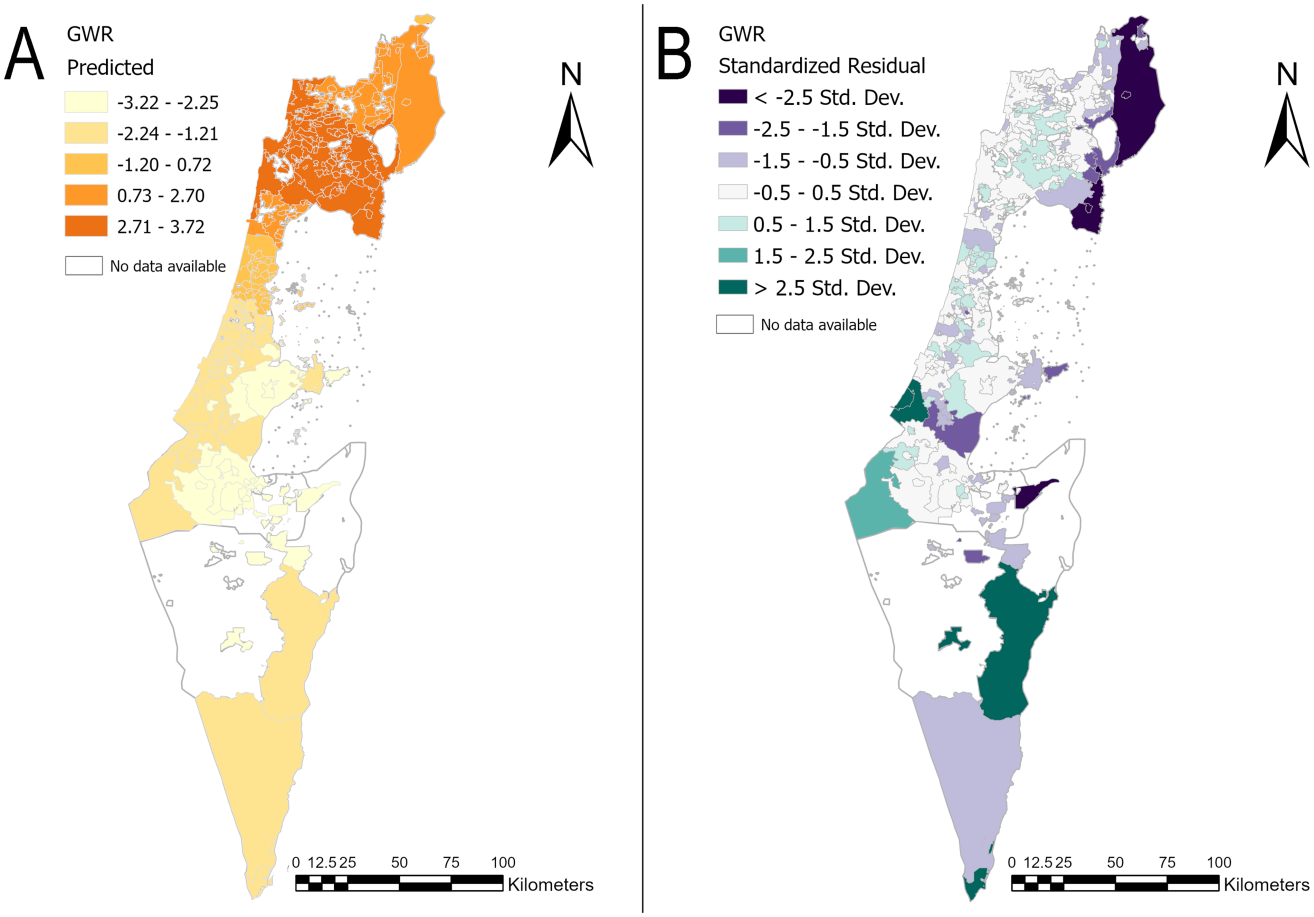
Prediction map and standardized residuals map of GWR analysis. **(A)** Map of predicted z-scores of childhood obesity by locality in 2014. **(B)** Standardized residuals map of the z-score childhood obesity by locality in 2014. The basemaps are reprinted from https://www.cbs.gov.il/EN/Pages/default.aspx under a CC BY license, with permission from the Central Bureau of Statistics (CBS) and the Ministry of Interior, State of Israel, original copyright (1997–2024).

Figure 4A presents the map of predicted z-scores of childhood obesity across the study area alongside the standardized residuals map for the GWR model. The comparison of the predicted values alongside the residuals can help validate the model. Accordingly, if areas with high predicted values align with observed high z-scores of childhood obesity and low residuals, it supports the model’s accuracy. The maps in Figure 4 shows that standardized residuals less than ±2 indicate relatively small differences between observed and predicted values, suggesting a good model fit in most geographic hotspots in the north and geographic coldspots clusters in the center of the country. Supplementary Figures S1, S2 represent the prediction map and standardized residuals map of GWR analysis for the 42,000 m distance band and 65,000 m distance band, respectively. Those maps were created for the sensitivity analysis and are available for comparison in Appendix.

Table 3 presents the percentage of significance of the explanatory variables according to the MGWR model. Four variables were found to be significant.

**Table 3:** Summary of explanatory variables and neighborhoods for MGWR model.

Explanatory variables	Significance (% of features)
Intercept (Scaled)	234 (100.00)
Average vehicle licence fee	0 (0.00)
Percent of recipients of income support and income supplement to old age pension	1 (0.43)
Average years of schooling, of aged 25–54	158 (67.52)
Percent of families with 4 or more children	234 (100.00)
Dependency ratio	0 (0.00)
Median age	0 (0.00)
Socio-economic index	234 (100.00)

The socioeconomic index and the percentage of families with four or more children contributed to the regression model across all geographic areas. Additionally, the average years of schooling for individuals aged 25–54 significantly contributed to the model in 67% of the localities. The parameters that were not found to be significant suggest that they do not have a substantial influence across the entire geographic area.

Table 4 represents the summary statistics of the coefficients that were found to be significant and their standard deviation. The average socioeconomic index coefficient is −0.89, with a range of −1.09 to −0.73. This consistent negative association across all geographic areas indicates that regions with lower socioeconomic indices are more likely to have higher childhood obesity z-scores, and vice versa. The average coefficient for the percentage of families with four or more children is 1.466, ranging from 1.453 to 1.498. This positive association with minimal variation suggests that areas with a higher percentage of families with four or more children tend to have higher childhood obesity z-scores, and vice versa. The average coefficient for years of schooling is 0.563, ranging from 0.08 to 0.77. This positive relationship suggests that an increase in the average years of schooling is associated with higher childhood obesity z-scores, although the effect size is smaller compared to the percentage of families with four or more children. It is also important to note that the average years of schooling and percentage of families with four or more children were standardized, as described in the methods section. Consequently, each coefficient represents a change in standard deviations, meaning even relatively small coefficients can result in significant changes.

**Table 4:** Summary statistics for coefficients estimates for the MGWR model.

Explanatory variables	Mean	Standard deviation	Minimum	Median	Maximum
Intercept (scaled)	0.038	0.259	−0.853	−0.141	0.446
Average years of schooling of those aged 25–54	0.563	0.095	0.08	0.548	0.77
Percent of families with 4 or more children	1.466	0.006	1.453	1.467	1.498
Socio-economic index	−0.878	0.073	−1.09	−0.879	−0.731

**Figure 5:**
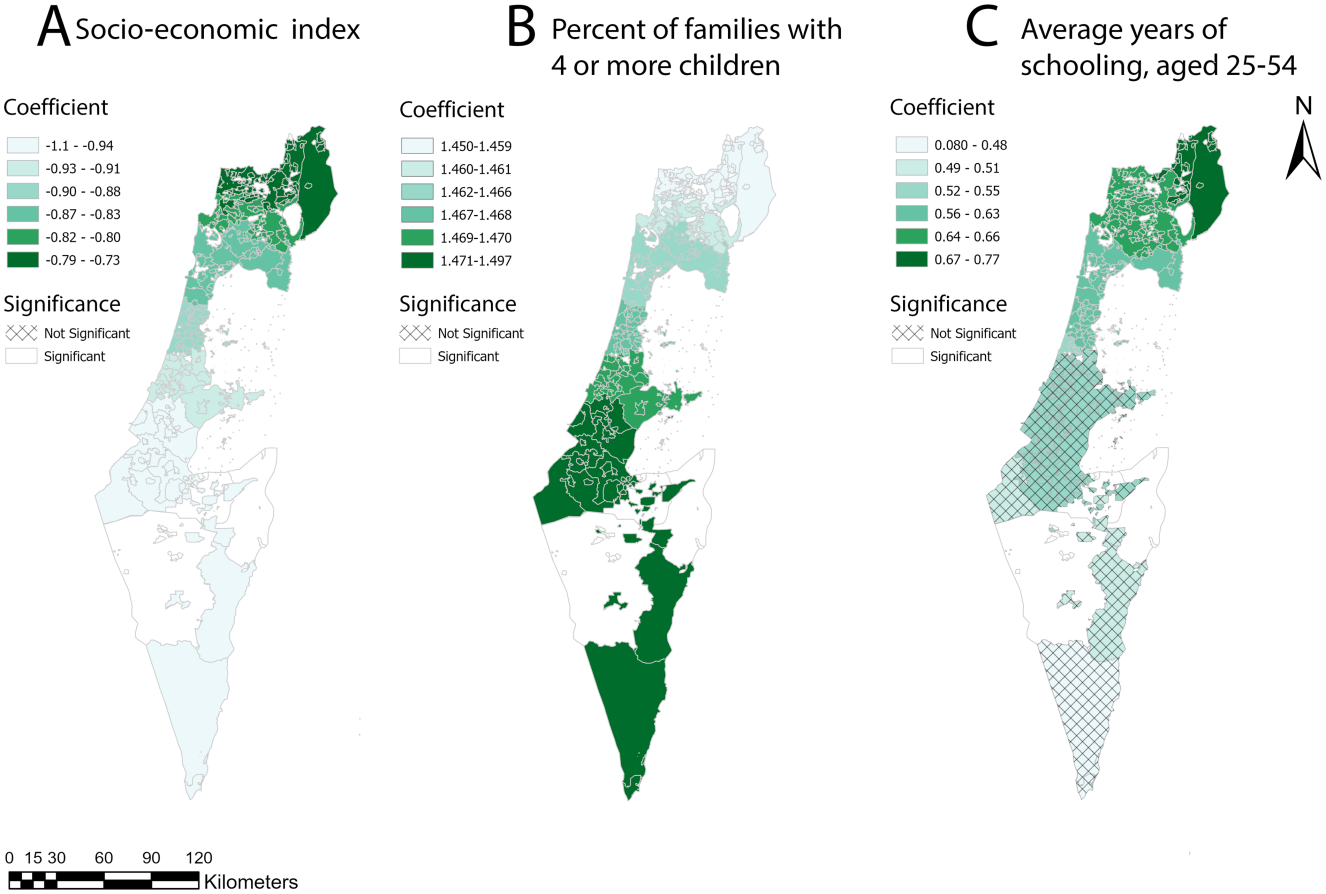
Maps of the regression analysis coefficients were created using the Getis-Ord $G_{i}^{*}\;$ statistic for 2014, based on a 51,000-meter bandwidth for the MGWR model. **(A)** A map of socio-economic index coefficient distribution by locality, based on the 51,000-meter distance band z-score. **(B)** A map of the percentage of families with 4 or more children coefficient distribution by locality, based on the 51,000 m distance band z-score. **(C)** A map of average years of schooling, aged 25–54 years coefficient distribution by locality, based on the 51,000 m distance band z-score.

This variable is significant in 67% of the localities. The distribution of significant coefficients is concentrated in the northern part of the country. These northern regions are identified as including hotspots and clusters of localities with a high z-score of childhood obesity. Figure 5 displays thematic maps of the regression coefficients. In Supplementary material, the implications of different distance bands were explored on the resulting maps derived from the Getis-Ord $G_{i}^{*}\;$ analysis and examined their effects on the GWR and MGWR outputs, including the coefficients. The socioeconomic index and the percentage of families with four or more children remain consistently significant across all geographic areas, showing the same negative and positive associations, respectively. However, the average years of schooling was not found to be significant at other distance bands. The thematic maps of the regression coefficients for the 42,000 m distance band are presented in Supplementary Figure S3, and those for the 65,000 m distance band are shown in Supplementary Figure S4.

The basemaps are reprinted from https://www.cbs.gov.il/EN/Pages/default.aspx under a CC BY license, with permission from the Central Bureau of Statistics (CBS) and the Ministry of Interior, State of Israel, original copyright (1997–2024).

## Discussion

The study explored the spatial patterns of childhood obesity in relation to socioeconomic and demographic factors. The median percentage of childhood obesity did not show a statistically significant difference between 2014 and 2018, indicating that the medians remained consistent over those years. However, spatial autocorrelation by Moran’s I showed that dependencies within localities are not random and that spatial patterns of childhood obesity z-scores exist. The prevalence maps of childhood obesity and hotspot analysis maps (Figures 2, 3) for 2014 and 2018 revealed changes in the central and northern regions of the country. Specifically, the sizes of both hotspot and coldspot clusters increased. This highlights that statistical analysis without accounting for spatial effects is insufficient to evaluate changes in childhood obesity patterns. Spatial dependency is a key factor in understanding the geographic patterns of childhood obesity, yet it is often overlooked in research. For instance, Rossman et al. (61) conducted a large-scale study involving 132,262 children in Israel to predict childhood obesity at ages 5–6. They employed a machine learning algorithm (gradient boosting trees) and incorporated geographic location to generate validation datasets for their prediction task. However, the method they used to represent geographic distribution was not thoroughly described, and no formal tests for spatial autocorrelation were conducted. Despite this, their model achieved strong predictive performance, with an area under the curve (AUC) of 0.8. It is possible that incorporating spatially dependent clusters based on explicit spatial autocorrelation could further enhance model accuracy (61).

Incorporating spatial analysis provides valuable insights into prevalence changes across different geographical areas. The geographical presentation that was identified in this study showed that while in one area there are more localities with low obesity prevalence (the central region), in the other area (the north) there were more localities with high obesity prevalence. Furthermore, the distribution of childhood obesity exhibited a high concentration of clusters in the peripheral areas of the country. The attached maps (Figures 2B, 3B) showed clusters of geographical areas with high obesity prevalence marked on the hotspot analysis maps in red and indicated statistical significance areas for childhood obesity with a safety margin of 99%. These red zones were particularly prominent in the northern region on the maps for 2014 and 2018. Hotspot analysis helped examine geographic areas that require targeted obesity interventions. In addition, red areas representing localities with high childhood obesity prevalence, which are adjacent to other localities with similarly high prevalence (as examined by z-scores of childhood obesity prevalence), should be further examined. These areas should be targeted for childhood health promotion programs aimed at mitigating the increasing prevalence of childhood obesity and subsequent morbidity. Results from differing interventional models at the central areas versus the peripheral areas should be compared in terms of efficacy and target population in order to further develop cost-effective, tailored approaches by population characteristics. Areas with the highest prevalence of obesity should be targeted for immediate interventions.

A negative relationship between socioeconomic status and childhood obesity in industrialized and developed areas is known, according to several studies (27, 28). However, the impact of clustering on childhood obesity has not been thoroughly investigated, and the findings regarding childhood obesity in developed societies are inconclusive. Consequently, the association between obesity and socioeconomic factors differs based on gender, age, and country (28–30). In the Middle East, higher socioeconomic status is associated with childhood obesity (20). Few studies examined this relationship however, the spatial dependency and heterogeneity were not taken into account. Al-Kloub et al. reported that adolescents living in areas with higher socioeconomic status (SES) in Amman, Jordan, were more likely to be overweight or obese. However, their cross-sectional study relied on logistic regression analysis, which assumes linearity and interdependence between variables. While sociodemographic parameters were analyzed, the study did not account for geographic relationships, and no spatial analysis was performed. As a result, the geographic influence on these parameters remains unclear (62). Similar findings showing a positive association between socioeconomic status and obesity were reported in Saudi Arabia by Ibrahim Al Alwan, using multivariate logistic regression analysis. However, the study did not account for spatial dependency in its analysis (63).

The study results exhibit an association between higher z-scores for childhood obesity prevalence and lower socioeconomic status (mean coefficient: −0.878, range: −1.09 to −0.731). The socioeconomic index was significant in all the study areas when analyzing the overall impact of parameters on variability and localized effects. Our findings of a negative association between the socioeconomic index and z-scores of childhood obesity are consistent with observations in industrialized areas but differ from the corresponding relationship reported in other parts of the Middle East (29). These results align with patterns observed in other high-income countries, such as Israel, and may contrast with trends in middle and low-income countries elsewhere in the Middle East (20). However, a positive effect was observed for the average years of schooling (mean coefficient: 0.563, range: 0.08 to 0.77). This result contrasts with findings from other high-income countries, where an inverse relationship between parental education and childhood overweight is typically observed. Instead, it aligns more closely with trends in middle-and low-income countries (29). Al-Kloub et al. found that higher paternal education in Amman, Jordan, was associated with an increased likelihood of adolescent overweight and obesity (62). In Saudi Arabia, maternal education showed a similar positive association with childhood overweight (63). While both studies examined parental education as an individual-level predictor, this research shifts the focus to the neighbourhood context, analysing spatial relationships to capture the broader local influence on childhood obesity. It is important to note that this relationship is observed at the community level and does not imply causation at the individual or family level. Instead, it may reflect broader socio-economic or cultural trends that warrant further investigation. This neighbourhood-based perspective offers an alternative public-health strategy that complements, rather than replaces, individual-level interventions.

The positive relationship between the average years of schooling and z-score of childhood obesity prevalence is significant in only 67% of the localities. The distribution of significant coefficients is concentrated in the northern part of the country. These northern regions are identified as including hotspots and clusters of localities with high z-score of childhood obesity. It is worth noting that the average years of schooling were not significant for the 42,000 m and 65,000 m bands. For the 65,000 m band, this variable exhibited both negative and positive coefficients but remained statistically insignificant. Its significance at the 51,000 m distance band may be due to the choice of this band, which optimally captures localized effects. The 51,000 m band corresponds to the maximum points where the z-score rises and then descends, making it particularly effective for identifying localized impacts of this variable on childhood obesity z-scores. Further studies are needed to examine this effect in greater detail to ensure the generalizability of these findings.

A positive average effect was observed for the percentage of families with four or more children across the entire geographic area (mean coefficient: 1.466, range: 1.453 to 1.498). This suggests that as the average number of children per family in a locality increases, the likelihood of that locality being included in a cluster of areas with high obesity rates also increases. Furthermore, the effect of the coefficients is observed not only in areas with low and high z-scores for childhood obesity but also in other areas that do not form clusters. This indicates that the variables influence all localities, not just those within clusters. To reinforce the credibility of these findings, sensitivity tests demonstrated that these two variables remain consistently significant across the entire area, with the same negative and positive associations.

Few studies examined the relationship between family size and childhood obesity (62, 64, 65). Data found in her research that having more siblings is associated with a lower likelihood of obesity. The study concluded that children with siblings have healthier diets and watch less television (65). Therefore, the association of larger families with more children with geographic clusters of higher obesity prevalence may imply an indirect relationship to lower socioeconomic index as an explanation for the obesogenic environment.

In the Middle East, the association between family size and childhood obesity remains inconclusive. Some studies report a negative relationship, while others report a positive one, and some find no significant association at all. However, these studies primarily focus on the individual level and lack community-level analysis or relevant geographic methods (62).

Another parameter that supported local variations in the relationship between the z-score of childhood obesity percentage and sociodemographic factors is the bandwidth of 66 neighbors. By using this number of neighbors, the model captured variations in these relationships across different localities within this spatial range. This approach helped identify specific areas where the association between the z-score of childhood obesity and sociodemographic factors differed, providing more detailed insights into the spatial heterogeneity of the studied phenomena.

The geographic context plays a significant role in shaping the prevalence of childhood obesity, and understanding these dynamics is crucial for effective intervention strategies (46, 47). The study findings emphasize the complex relationship between childhood obesity and environmental factors, while also highlighting spatial variability in explanatory variables and their localized effects. In addition, the inclusion of the localized socioeconomic and demographic factors provided new insights into how neighborhood-level characteristics may influence childhood obesity, rather than just individual-level efforts, and may help to uncover area-based relationships. The association between lower socioeconomic status and clusters of geographic areas with high prevalence of childhood obesity may imply a gap in childhood obesity prevalence between high and low socioeconomic groups (64). However, it is important to mention that this relationship is observed at the community level and does not imply causation at the individual or family level. It may reflect broader socio-economic or cultural trends that warrant further investigation.

### Limitations

In this study, the predicted values were compared with the standardized residuals, demonstrating that small, standardized residuals indicate small differences between the observed and predicted values. This suggests a good model fit in most geographic hotspots in the north and cold spots in the center of the country. However, this is not an absolute result. Some geographic hotspots and coldspots showed high standardized residuals, indicating that the model does not accurately predict outcomes for all localities. This suggests that the model does not capture all the underlying factors influencing z-scores of childhood obesity prevalence, or that the relationships between the variables may vary more than the model accounts for. Fixed-distance bands provide a straightforward approach to analyzing spatial relationships and can yield valuable insights into localized spatial patterns in studies where a consistent spatial scale is appropriate or computational efficiency is a concern. However, it is important to note that variable distance thresholds and scaling in spatial autocorrelation modelling have the potential to capture the spatial dynamics of complex geographical systems (47).

Additional limitations of the study relate to the selection and inclusion of predictor variables in the multivariable regression model. In particular, the inclusion of an income measure both within the socioeconomic index and as an individual variable may have introduced redundancy and collinearity. To mitigate this issue, variables exhibiting high correlation were excluded to minimize multicollinearity, as detailed in the methods section. Another important limitation to mention is that the z-scores derived from the Getis-Ord Gi* analysis carry inherent uncertainty, which was not explicitly accounted for in the GWR analysis. However, the original data used for this analysis, such as the average obesity prevalence for each locality, are also estimates and subject to variability. The use of z-scores allowed us to standardize and account for comparability across localities, ensuring that the analysis captured relative clustering patterns effectively. In addition, it is important to note that alternative spatial regression models are available. For example, Conditional Autoregression (CAR) models. These models use a spatial random effects approach to analyze the relationship between structured data (geographical clusters) and covariates. These models provide valuable insights into both global and localized effects of covariates and are often applied to small geographic areas, like the region studied in this research. However, the focus was placed on the GWR and MGWR models because of their simplicity and ease of interpretation. These models demonstrated a strong fit for exploring the relationship between z-scores of childhood obesity and socioeconomic and demographic factors.

Observing obesity prevalence at the neighborhood level can be used to identify subgroups within neighborhoods and communities, further enabling the establishment of targeted health promotion programs at the community level. Our study focused on socioeconomic index and included six more socioeconomic and demographic variables (median age, dependency ratio, percent of families with four children or more, average years of education of persons aged 25–54, percent of recipients of income support and income supplement to old age pension, average vehicle license fee).

Other demographic, social, and economic parameters may also play a significant role in shaping the obesogenic environment for children. Additionally, factors related to the physical and built environment were not evaluated for their potential impact on the association between childhood obesity clusters and socioeconomic factors. Urban exposome factors, such as green spaces and air quality, were also not directly considered in this study. However, the average license fee for each locality was included, which may provide indirect insights into the influence of the urban environment and partially reflect aspects of the urban exposome. To gain a more comprehensive understanding of these relationships, further studies incorporating these additional factors are needed to explore their connection with childhood obesity more thoroughly.

## Conclusion

Obesity is a global challenge and may be influenced by several modifiable environmental factors. A spatial-geographical approach can be effective for managing obesity at the population level by identifying environmental factors that influence human behavior and social dynamics (represented collectively in our study by socioeconomic status parameters). This approach serves as a valuable tool for recognizing and modifying existing norms and behavior patterns. The study identified a positive spatial clustering of high childhood obesity prevalence, suggesting that childhood obesity tends to cluster in geographical areas on the peripheries of Israel. The spatial approach allowed the identification of specific regions as hot spots for targeting and intervention. This is the first study to explore the relationship between childhood obesity cluster patterns in the Middle East and their association with socioeconomic factors. Furthermore, it sheds light on the patterns of childhood obesity clusters and aids in understanding how inequalities in regional economic status impact childhood obesity.

This study demonstrates a significant association between higher z-scores for childhood obesity prevalence and lower socioeconomic status across all geographic areas. This pattern is consistent with findings from industrialized regions but not with trends observed in other parts of the Middle East. The results also align with patterns seen in other high-income countries but may contrast with trends found in middle-and low-income countries elsewhere in the region.

A higher percentage of families with four or more children is associated with increased childhood obesity z-scores across the entire study area. Additionally, an association was found between higher z-scores and higher average years of schooling in 70% of localities located in the country’s periphery. This contrasts with findings from other high-income countries and more closely resembles trends observed in middle-and low-income nations.

These associations are observed at the community level and do not imply causation at the individual or family level. Rather, they may reflect broader socioeconomic or cultural dynamics that warrant further exploration. Further research is needed to determine the most effective strategies for addressing and reducing childhood obesity.

## Data Availability

The original contributions presented in the study are included in the article/Supplementary material, further inquiries can be directed to the corresponding author.
