# Supplementary material for: Spatial patterns of childhood obesity clusters linked to socioeconomic inequalities
Source: Front Public Health. 2025 Aug 19;13:1497090. doi: 10.3389/fpubh.2025.1497090 (PMC12404039; doi:10.3389/fpubh.2025.1497090)

**Appendix. Sensitivity analysis for distance band.**

We performed a sensitivity analysis to investigate how different distance bands influence the results of GWR and MGWR models. Specifically, we evaluated three distance bands: 42,000 meters (first peak), 51,000 meters (second peak), and 65,000 meters (third peak). These bands were selected based on the Moran's Index analysis, as shown in Figure 1 of the main text. To explore the implications, we compared the resulting maps from the Getis-Ord $G_{i}^{*}$analysis and examined their effects on the GWR and MGWR outputs. The findings are presented in Figures S1 and S2, which provide a direct comparison of the distance bands alongside the Getis-Ord $G_{i}^{*}$results.

Choosing an appropriate distance band depends heavily on the research objectives. The 51,000 meters band (second peak) was particularly effective, capturing broad spatial patterns such as variations between the north, center, and south regions while remaining sensitive to the local community level. A statistical comparison of the results across the three distance bands is available in Table S4.

**Table S4**. Statistical results of spatial regression models.

| Statistic | GWR  42,000 meters distance band | GWR  51,000 meters distance band | GWR  65,000 meters distance band |
| --- | --- | --- | --- |
| R-Squared | 0.95 | 0.97 | 0.98 |
| Adjusted R-Squared | 0.93 | 0.95 | 0.98 |
| AICc | 134.26 | 32.38 | 340.63 |
| Sigma-Squared | 0.07 | 0.05 | 0.15 |
| Sigma-Squared MLE | 0.05 | 0.03 | 0.09 |
| Effective Degrees of Freedom | 151.44 | 155.02 | 136.56 |

The GWR model for the 51,000 meters distance band had the lowest AICc value (32.3) compared to the models for the 42,000 meters band (134.26) and the 65,000 meters band (340.63). This lower AICc suggests that the 51,000 meters distance band model more effectively captures spatial variation and explains a greater portion of the variance in the data compared to the other distance bands.

All models demonstrated high R-squared and adjusted R-squared values, ranging between 0.93 and 0.98. Although the 65,000 meters band showed a slightly higher adjusted R-squared (0.98) than the 51,000 meters band (0.95), the difference was minimal. However, the 65,000 meters band model also had a higher AICc and sigma-squared value, suggesting a less efficient model fit. Additionally, the 51,000 meters band model had higher effective degrees of freedom (155.02) compared to the models for the 42,000 meters band (151.44) and the 65,000 meters band (136.56). This suggests that the 51,000 meters band model captures more localized spatial variation.

Figures S2 and S3 illustrate the thematic maps for the 42,000 meters and 65,000 meters distance bands for 2014, respectively. The thematic maps for the 51,000 meters distance band are included in the main text.

**Figure S1.** Hotspot analysis map, prediction map and standardized residuals map of General Weighted Regression analysis for 2014- and 42,000-meters distance band.


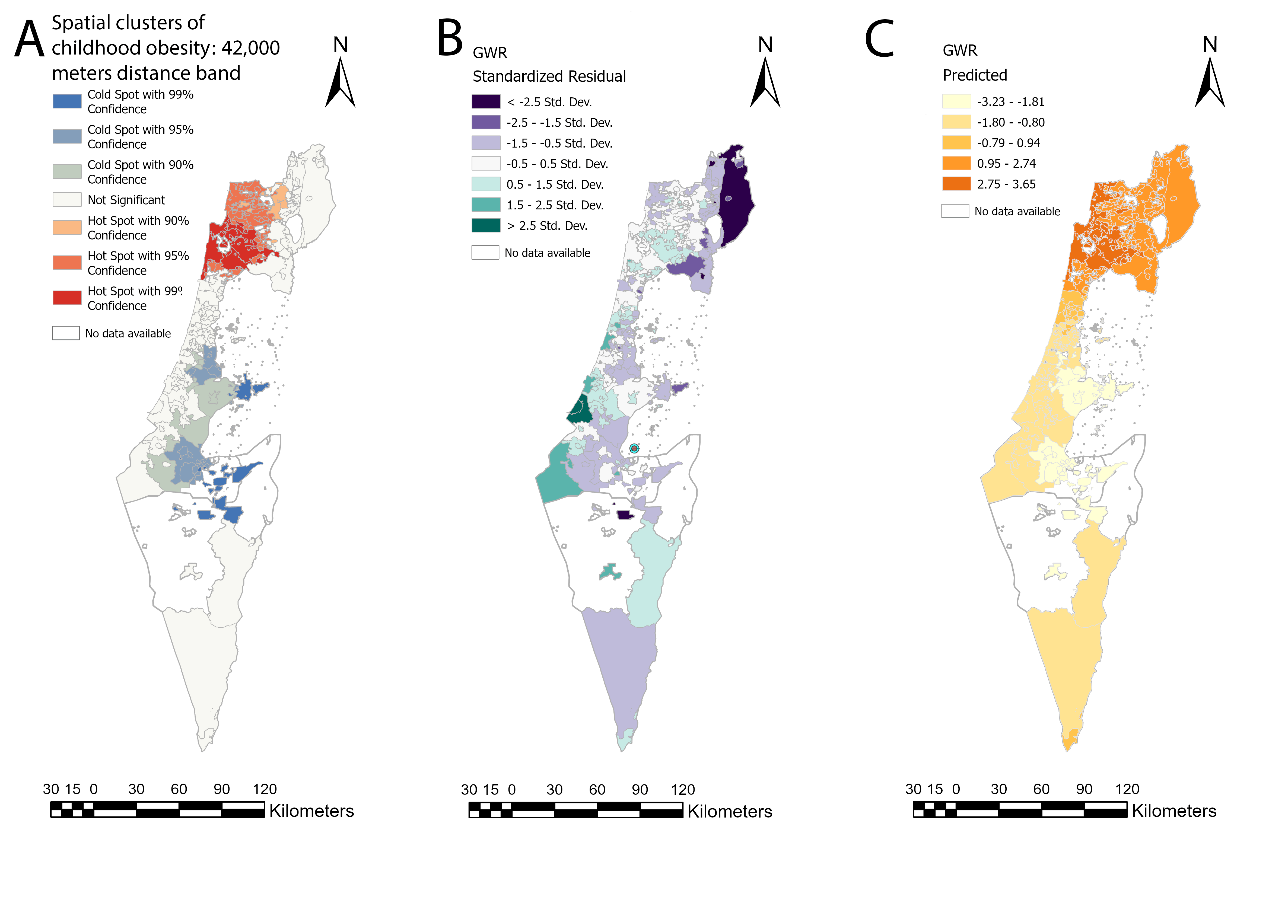


(A) A hotspot analysis map of localities with childhood obesity in 2014, based on the 42,000 meters distance band. (B) A map of predicted z-scores for childhood obesity by locality in 2014, derived from the 42,000 meters distance band hotspot analysis. (C) A map of standardized residuals for z-scores of childhood obesity by locality in 2014, based on the 42,000 meters distance band hotspot analysis. The basemaps reprinted from https://www.cbs.gov.il/EN/Pages/default.aspx under a CC BY license, with permission from the Central Bureau of Statistics(CBS) and the Ministry of Interior, State of Israel, original copyright [1997-2024].

**Figure S2.** Hotspot analysis map, prediction map and standardized residuals map of General Weighted Regression analysis for 2014- and 65,000-meters distance band.


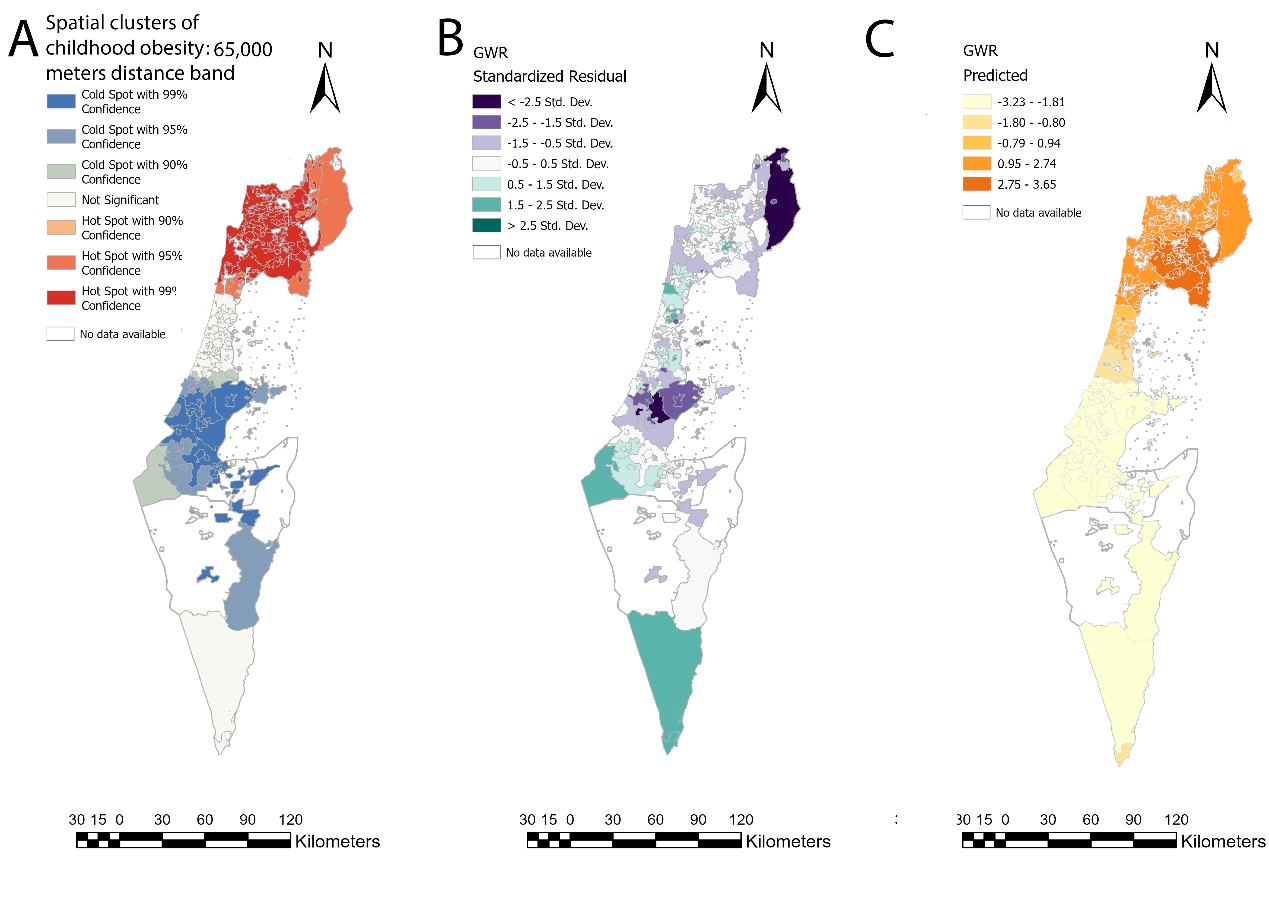


1. A hotspot analysis map of localities with childhood obesity in 2014, based on the 65,000 meters distance band. (B) A map of predicted z-scores for childhood obesity by locality in 2014, derived from the 65,000 meters distance band hotspot analysis. (C) A map of standardized residuals for z-scores of childhood obesity by locality in 2014, based on the 65,000 meters distance band hotspot analysis.

The basemaps reprinted from https://www.cbs.gov.il/EN/Pages/default.aspx under a CC BY license, with permission from the Central Bureau of Statistics(CBS) and the Ministry of Interior, State of Israel, original copyright [1997-2024].

We also examined how the choice of distance band affects the coefficients of the explanatory variables. Table S5 summarizes the comparison of the significance of key variables across the three distance bands. The analysis focused on three major variables: socioeconomic index, percentage of households with four or more children, and average years of schooling. The two variables: socioeconomic index and percentage of households with four or more children remained consistently significant across all three distance bands and across the entire geographic area. The maps indicate slight variations in the spatial distribution of these coefficients. Both variables maintained the same relationships: a negative association for the socioeconomic index and a positive association for the percentage of households with four or more children. However, the percentage of households with four or more children showed small local variation in its coefficients across the three analyses. The third variable, average years of schooling, was not significant for the 42,000 meters and 65,000 meters bands. For the 65,000 meters band, this variable exhibited both negative and positive coefficients but remained statistically insignificant. As a result, these changes in the coefficients had no meaningful implications, and no conclusions could be drawn from this change.

The coefficients for each distance band are illustrated in the following figures: Figure S3 for the 42,000 meters band, and Figure S4 for the 65,000 meters band. The coefficients for the 51,000 meters band are presented in Figure 6 in the main text.

Table S6 summarizes statistics for coefficients estimates between the explanatory variables for the three different distance bands.

**Table S5.** Comparison between the significance of the explanatory variables between the three distance bands for the MGWR.

| Explanatory Variables | Significance 42,000 meters band(% of Features)^b^ | Significance 51,000 meters band(% of Features)^b^ | Significance 65,000 meters band(% of Features)^b^ |
| --- | --- | --- | --- |
| Intercept (Scaled) | 169 (72.22) | 234 (100.00) | 180 (76.92) |
| Average vehicle licence fee | 0 (0.00) | 0 (0.00) | 0 (0.00) |
| Percent of recipients of income support and income supplement to old age pension | 1 (0.43) | 1 (0.43) | 4 (1.71) |
| Average years of schooling, of aged 25-54 | 5 (2.14) | 158 (67.52) | 0 (0.00) |
| Percent of families with 4 or more children | 234 (100.00) | 234 (100.00) | 234 (100.00) |
| Dependency ratio | 0 (0.00) | 0 (0.00) | 0 (0.00) |
| Median age | 0 (0.00) | 0 (0.00) | 0 (0.00) |
| Socio-economic index | 234 (100.00) | 234 (100.00) | 234 (100.00) |

**Table S6.** Summary statistics for coefficients estimates. Comparison between the explanatory variables between the three distance bands for the MGWR model.

| Explanatory Variables | |  | Mean |  |  | Standard Deviation |  |  | Minimum |  |  | Maximum |  |
| --- | --- | --- | --- | --- | --- | --- | --- | --- | --- | --- | --- | --- | --- |
| Distance band (meters) | | **42,000** | **51,000** | **65,000** | **42,000** | **51,000** | **65,000** | **42,000** | **51,000** | **65,000** | **42,000** | **51,000** | **65,000** |
| Intercept (Scaled) | 0.039 | 0.038 | 0.034 | 0.252 | 0.259 | 0.284 | -0.852 | -0.853 | -1.008 | 0.432 | 0.446 | 0.479 |  |
| Average vehicle licence fee | | 0.019 | 0.126 | 0.027 | 0.021 | 0.103 | 0.024 | -0.029 | -0.066 | -0.04 | 0.171 | 1.038 | 0.079 |
| Income support^a^ | | -0.0007 | 0.090 | 0.062 | 0.037 | 0.094 | 0.049 | -0.028 | 0.012 | 0.016 | 0.245 | 0.694 | 0.361 |
| Years of schooling^b^ | | 0.166 | 0.563 | 0.097 | 0.039 | 0.095 | 0.048 | 0.014 | 0.08 | -0.064 | 0.262 | 0.77 | 0.204 |
| Number of children ^c^ | | 0.584 | 1.466 | 0.53 | 0.003 | 0.006 | 0.012 | 0.579 | 1.453 | 0.507 | 0.599 | 1.498 | 0.607 |
| Dependency ratio | | 0.077 | 0.182 | 0.098 | 0.002 | 0.005 | 0.002 | 0.074 | 0.172 | 0.092 | 0.087 | 0.208 | 0.102 |
| Median age | | -0.054 | -0.011 | -0.01 | 0.012 | 0.009 | 0.012 | -0.084 | -0.05 | -0.05 | -0.05 | 0.007 | .013 |
| Socio-economic index | | -0.695 | -0.878 | -0.7 | 0.035 | 0.073 | 0.027 | -0.746 | -1.09 | -0.75 | -0.446 | -0.731 | -0.587 |

Notes; **^a^** :Percent of recipients of income support and income supplement to old age pension. **^b^**:Average years of schooling, of aged 25-54. **^c^**:Percent of families with 4 or more children.

Figure S3. Maps of the coefficients of the 42,000 meters distance band MGWR.


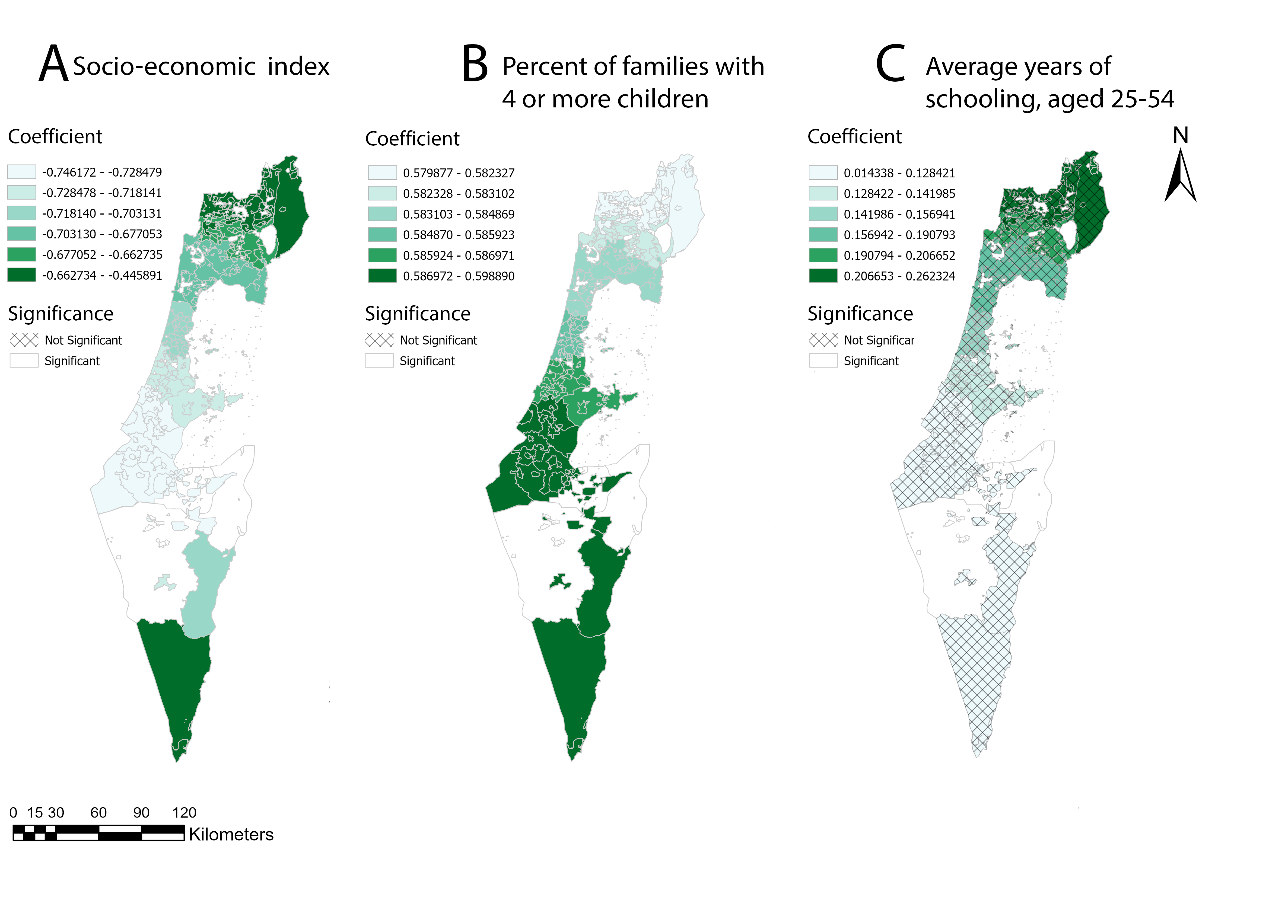


1. A map of socio-economic index coefficient distribution by locality, based on the 42,000 meters distance band z-score. (B A map of percent of families with 4 or more children coefficient distribution by locality, based on the 42,000 meters distance band z-score. (C) A map of average years of schooling, aged 25-54 years coefficient distribution by locality, based on the 42,000 meters distance band z-score.

The basemaps reprinted from https://www.cbs.gov.il/EN/Pages/default.aspx under a CC BY license, with permission from the Central Bureau of Statistics(CBS) and the Ministry of Interior, State of Israel, original copyright [1997-2024].

Figure S4. Maps of the coefficients of the 65,000 meters distance band MGWR.


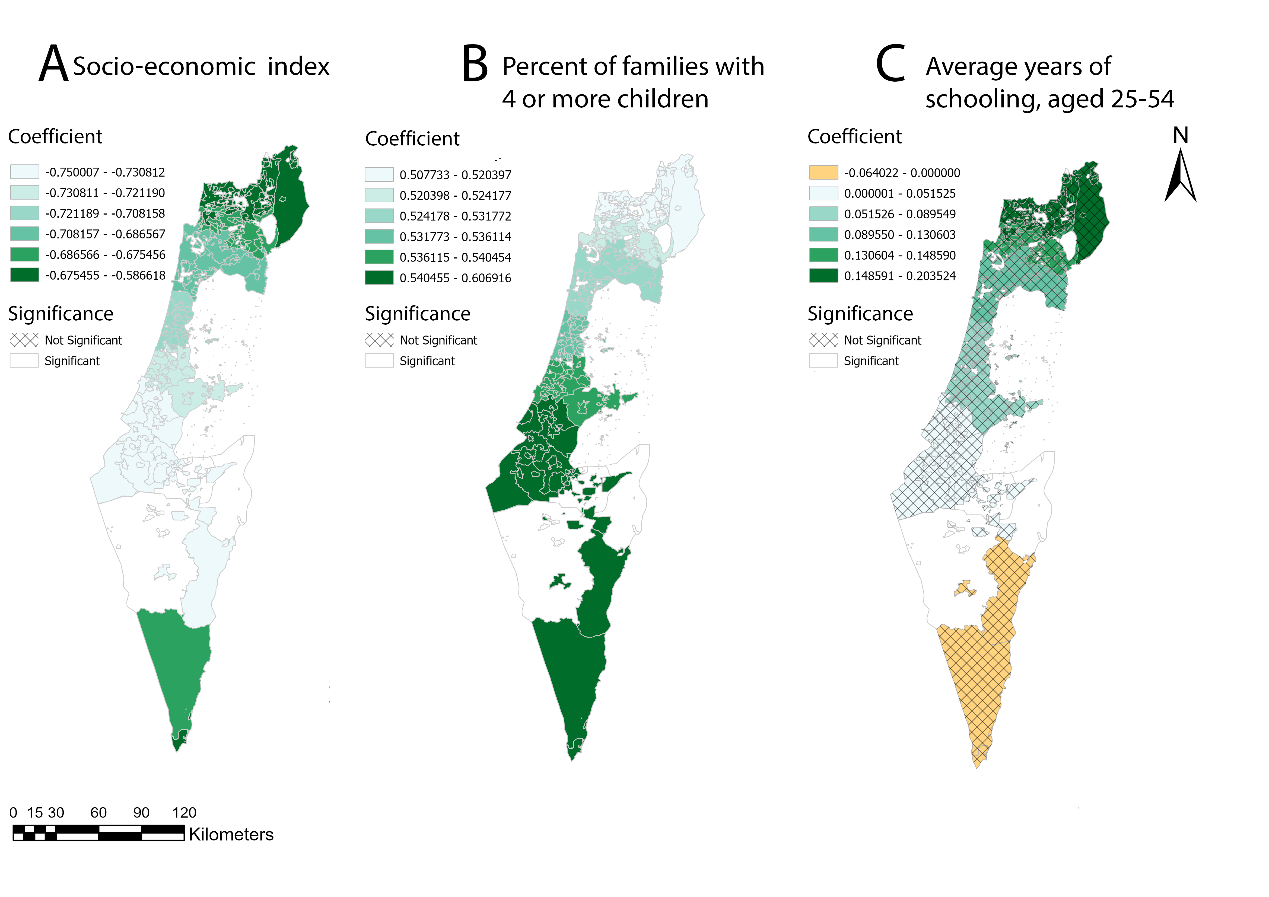


1. A map of socio-economic index coefficient distribution by locality, based on the 65,000 meters distance band z-score. (B A map of percent of families with 4 or more children coefficient distribution by locality, based on the 65,000 meters distance band z-score. (C) A map of average years of schooling, aged 25-54 years coefficient distribution by locality, based on the 65,000 meters distance band z-score.

The basemaps reprinted from https://www.cbs.gov.il/EN/Pages/default.aspx under a CC BY license, with permission from the Central Bureau of Statistics(CBS) and the Ministry of Interior, State of Israel, original copyright [1997-2024].

The first Moran I test(10,000 meters interval up to a range of 300,000 meters) was exhibited highest peak at 51,000 meters as represents by Figure S6.

Figure S5. First Moran's index analysis for a vast geographic area that encompassed the entire country.


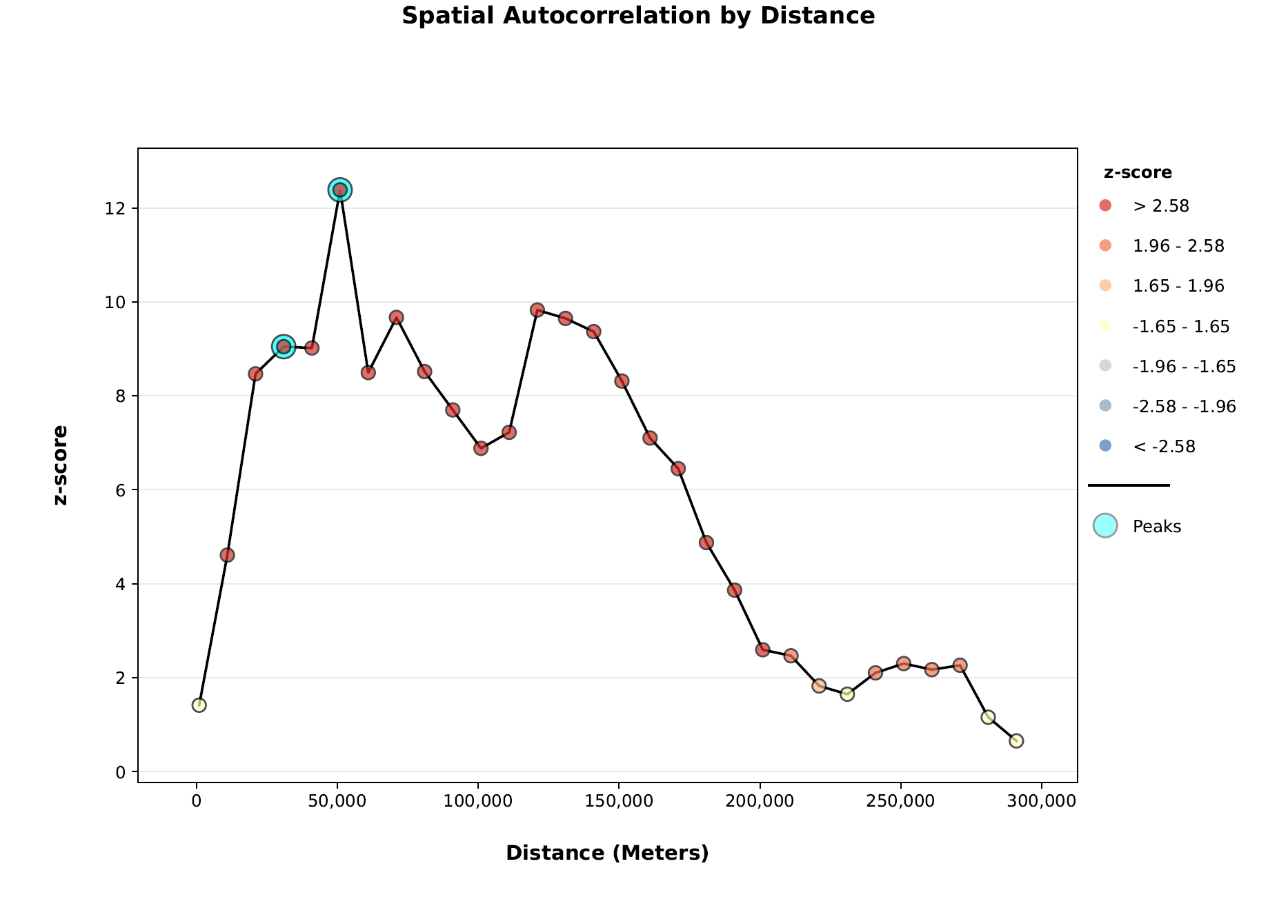

Supplement: Supplementary file 1 [file Data_Sheet_1.docx]
