# Supplementary material for: Spatial patterns of childhood obesity clusters linked to socioeconomic inequalities
Source: Front Public Health. 2025 Aug 19;13:1497090. doi: 10.3389/fpubh.2025.1497090 (PMC12404039; doi:10.3389/fpubh.2025.1497090)
Supplement: Supplementary file 2 [file Table_1.docx]

**S1 Table. Hot spot analysis results for 2014.**

| **Locality Name** | **Obesity percentage** | **GiZScore** | **GiPValue** | **NNeighbors** | | **Gi_Bin** |
| --- | --- | --- | --- | --- | --- | --- |
| EMEQ YIZRE'EL | 0.08 | 3.77337 | 0.000161 | 109 | 3 | |
| Zevulun | 0.08 | 3.680669 | 0.000233 | 107 | 3 | |
| MEGIDDO | 0.02 | 3.437855 | 0.000586 | 114 | 3 | |
| Iksal | 0.06 | 3.738673 | 0.000185 | 109 | 3 | |
| AL-BATOF | 0.07 | 3.367983 | 0.000757 | 107 | 3 | |
| I'BILLIN | 0.13 | 3.611183 | 0.000305 | 107 | 3 | |
| BUSTAN EL-MARJ | 0.07 | 3.431804 | 0.0006 | 108 | 3 | |
| BU'EINE-NUJEIDAT | 0.06 | 3.072907 | 0.00212 | 104 | 3 | |
| BIR EL-MAKSUR | 0.04 | 3.738673 | 0.000185 | 109 | 3 | |
| BASMAT TAB'UN | 0.07 | 3.640116 | 0.000273 | 108 | 3 | |
| JUDEIDE-MAKER | 0.13 | 3.449664 | 0.000561 | 100 | 3 | |
| Julis | 0.1 | 3.099794 | 0.001937 | 100 | 3 | |
| DALIYAT AL-KARMEL | 0.14 | 4.213579 | 2.51E-05 | 111 | 3 | |
| DABURIYYA | 0.11 | 3.530491 | 0.000415 | 109 | 3 | |
| DEIR HANNA+A5 | 0.07 | 3.114125 | 0.001845 | 103 | 3 | |
| ZIKHRON YA'AQOV | 0.06 | 3.044733 | 0.002329 | 109 | 3 | |
| Zarzir | 0.07 | 3.605397 | 0.000312 | 108 | 3 | |
| HOF HAKARMEL | 0.08 | 3.33324 | 0.000858 | 107 | 3 | |
| Haifa | 0.1 | 3.853738 | 0.000116 | 102 | 3 | |
| Tirat Karmel | 0.15 | 3.886543 | 0.000102 | 98 | 3 | |
| Tamra | 0.08 | 3.478166 | 0.000505 | 106 | 3 | |
| YAFI | 0.11 | 3.605397 | 0.000312 | 108 | 3 | |
| YOQNE'AM ILLIT | 0.09 | 4.161009 | 3.17E-05 | 115 | 3 | |
| Kabul | 0.11 | 3.379992 | 0.000725 | 105 | 3 | |
| KAOKAB ABU AL-HIJA | 0.05 | 3.367983 | 0.000757 | 107 | 3 | |
| KA'ABIYYE-TABBASH-HAJAJRE | 0.07 | 3.605397 | 0.000312 | 108 | 3 | |
| KAFAR YASIF | 0.11 | 3.099794 | 0.001937 | 100 | 3 | |
| KAFAR KANNA | 0.09 | 3.53596 | 0.000406 | 108 | 3 | |
| KAFAR MANDA | 0.08 | 3.53596 | 0.000406 | 108 | 3 | |
| MAJD AL-KURUM | 0.11 | 2.987866 | 0.002809 | 101 | 3 | |
| MIGDAL HAEMEQ | 0.13 | 3.77337 | 0.000161 | 109 | 3 | |
| Misgav | 0.08 | 3.114125 | 0.001845 | 103 | 3 | |
| MESHHED | 0.13 | 3.530491 | 0.000415 | 109 | 3 | |
| NAZERAT ILLIT | 0.13 | 3.530491 | 0.000415 | 109 | 3 | |
| NAZARETH | 0.11 | 3.664 | 0.000248 | 110 | 3 | |
| Nesher | 0.15 | 3.734705 | 0.000188 | 104 | 3 | |
| SAKHNIN | 0.12 | 3.114125 | 0.001845 | 103 | 3 | |
| ISIFYA | 0.1 | 4.312567 | 1.61E-05 | 112 | 3 | |
| EILABUN | 0.18 | 3.114125 | 0.001845 | 103 | 3 | |
| Ilut | 0.04 | 3.664 | 0.000248 | 110 | 3 | |
| AKKO | 0.15 | 3.120758 | 0.001804 | 102 | 3 | |
| ARRABE | 0.12 | 3.114125 | 0.001845 | 103 | 3 | |
| FUREIDIS | 0.13 | 3.48439 | 0.000493 | 105 | 3 | |
| QIRYAT BIALIK | 0.1 | 3.560548 | 0.00037 | 104 | 3 | |
| QIRYAT TIV'ON | 0.08 | 4.050946 | 5.1E-05 | 109 | 3 | |
| QIRYAT YAM | 0.19 | 3.393055 | 0.000691 | 103 | 3 | |
| REINE | 0.1 | 3.629323 | 0.000284 | 110 | 3 | |
| REKHASIM | 0.05 | 3.715412 | 0.000203 | 107 | 3 | |
| Ramat Yishay | 0.07 | 3.976102 | 7.01E-05 | 110 | 3 | |
| SHIBLI-UMM AL-GHANAM | 0.09 | 3.530491 | 0.000415 | 109 | 3 | |
| Sha'ab | 0.13 | 3.560548 | 0.00037 | 104 | 3 | |
| SHEFAR'AM | 0.13 | 3.605397 | 0.000312 | 108 | 3 | |
| Afula | 0.13 | 3.688914 | 0.000225 | 112 | 3 | |
| QIRYAT ATTA | 0.15 | 3.762784 | 0.000168 | 105 | 3 | |
| QIRYAT MOTZKIN | 0.14 | 3.637119 | 0.000276 | 103 | 3 | |
| ALLONA | 0.08 | 2.887815 | 0.003879 | 113 | 2 | |
| ABU SINAN | 0.11 | 2.939344 | 0.003289 | 98 | 2 | |
| UMM AL-FAHM | 0.13 | 2.728535 | 0.006362 | 119 | 2 | |
| BEIT JANN | 0.07 | 2.525363 | 0.011558 | 97 | 2 | |
| BI'NE | 0.11 | 2.67337 | 0.007509 | 101 | 2 | |
| JISR AZ-ZARQA | 0.23 | 2.216996 | 0.026623 | 91 | 2 | |
| JISH(GUSH HALAV) | 0.13 | 2.323507 | 0.020152 | 91 | 2 | |
| DEIR AL-ASAD | 0.02 | 2.679949 | 0.007363 | 100 | 2 | |
| Hagilbo'a | 0.1 | 2.527393 | 0.011491 | 102 | 2 | |
| Hagalil Hatahton | 0.04 | 2.77172 | 0.005576 | 102 | 2 | |
| Hurfeish | 0.07 | 2.688886 | 0.007169 | 94 | 2 | |
| TIBERIAS | 0.12 | 2.814073 | 0.004892 | 96 | 2 | |
| TUBA-ZANGARIYYE | 0.07 | 2.285608 | 0.022277 | 87 | 2 | |
| Tur'an | 0.09 | 2.753605 | 0.005894 | 105 | 2 | |
| YANUH-JAT | 0.07 | 2.701027 | 0.006913 | 97 | 2 | |
| Yavne'el | 0.06 | 2.2443 | 0.024813 | 97 | 2 | |
| Yirka | 0.07 | 2.826881 | 0.0047 | 99 | 2 | |
| KISRA-SUMEI | 0.06 | 2.567763 | 0.010236 | 96 | 2 | |
| KEFAR WERADIM | 0.06 | 2.434276 | 0.014922 | 95 | 2 | |
| KAFAR KAMA | 0.12 | 2.829087 | 0.004668 | 104 | 2 | |
| KAFAR QARA | 0.14 | 2.420188 | 0.015512 | 109 | 2 | |
| Kefar Tavor | 0.08 | 2.916325 | 0.003542 | 107 | 2 | |
| KARMI'EL | 0.12 | 2.800328 | 0.005105 | 103 | 2 | |
| Mazra'a | 0.16 | 2.645743 | 0.008151 | 95 | 2 | |
| MATTE ASHER | 0.1 | 2.778886 | 0.005455 | 96 | 2 | |
| Ma'ale Yosef | 0.15 | 2.62629 | 0.008632 | 93 | 2 | |
| MA'ALOT-TARSHIHA | 0.08 | 2.343348 | 0.019112 | 93 | 2 | |
| Merom Hagalil | 0.06 | 2.512363 | 0.011993 | 94 | 2 | |
| Nahef | 0.11 | 2.791848 | 0.005241 | 99 | 2 | |
| Sajur | 0.05 | 2.658693 | 0.007844 | 98 | 2 | |
| FASSUTA | 0.16 | 2.688886 | 0.007169 | 94 | 2 | |
| PEQI'IN (BUQEI'A) | 0.16 | 2.419964 | 0.015522 | 97 | 2 | |
| ZEFAT | 0.07 | 2.477059 | 0.013247 | 94 | 2 | |
| RAME | 0.14 | 2.679949 | 0.007363 | 100 | 2 | |
| Shelomi | 0.19 | 2.771381 | 0.005582 | 85 | 2 | |
| Basma | 0.13 | 2.530298 | 0.011397 | 116 | 2 | |
| Mughar | 0.06 | 2.743258 | 0.006083 | 101 | 2 | |
| MA'ALE IRON | 0.11 | 2.763156 | 0.005725 | 119 | 2 | |
| NAHARIYYA | 0.14 | 2.687358 | 0.007202 | 90 | 2 | |
| Ar'ara | 0.09 | 2.801855 | 0.005081 | 109 | 2 | |
| HAZOR HAGELILIT | 0.05 | 2.09035 | 0.036586 | 89 | 1 | |
| Mevo'ot Hahermon | 0.07 | 1.860255 | 0.062849 | 78 | 1 | |
| EMEQ HAYARDEN | 0.07 | 1.92586 | 0.054122 | 92 | 1 | |
| QIRYAT SHEMONA | 0.19 | 1.992581 | 0.046307 | 48 | 1 | |
| EMEQ HEFER | 0.07 | 0.429389 | 0.66764 | 94 | 0 | |
| Golan | 0.06 | 0.401203 | 0.688271 | 55 | 0 | |
| Lev Hasharon | 0.09 | 0.535414 | 0.592364 | 86 | 0 | |
| SEDOT NEGEV | 0.13 | -1.48308 | 0.138054 | 34 | 0 | |
| Even Yehuda | 0.08 | 0.288211 | 0.773185 | 85 | 0 | |
| Or Yehuda | 0.1 | -1.79469 | 0.072703 | 93 | 0 | |
| OR AQIVA | 0.13 | 1.682174 | 0.092535 | 97 | 0 | |
| Azor | 0.09 | -1.54637 | 0.122014 | 91 | 0 | |
| Ashdod | 0.1 | -1.78211 | 0.074731 | 75 | 0 | |
| Eshkol | 0.07 | -1.14902 | 0.250548 | 21 | 0 | |
| ASHQELON | 0.12 | 0.414214 | 0.678718 | 53 | 0 | |
| BAQA AL-GHARBIYYE | 0.12 | 1.348686 | 0.177438 | 108 | 0 | |
| BUQ'ATA | 0.08 | 0.58898 | 0.555874 | 22 | 0 | |
| BET DAGAN | 0.11 | -1.72386 | 0.084734 | 92 | 0 | |
| BET SHE'AN | 0.13 | 0.658426 | 0.510264 | 70 | 0 | |
| BENE BERAQ | 0.04 | -1.01517 | 0.310024 | 92 | 0 | |
| Bat Yam | 0.15 | -1.65292 | 0.098348 | 90 | 0 | |
| JALJULYE | 0.15 | -0.3204 | 0.748665 | 97 | 0 | |
| JATT | 0.14 | 1.361634 | 0.173314 | 105 | 0 | |
| GIV'AT SHEMU'EL | 0.08 | -1.58212 | 0.113623 | 92 | 0 | |
| Giv'atayim | 0.1 | -1.65299 | 0.098334 | 92 | 0 | |
| GAN RAWE | 0.05 | -1.68873 | 0.09127 | 89 | 0 | |
| GANNE TIQWA | 0.07 | -1.7594 | 0.07851 | 91 | 0 | |
| DEROM HASHARON | 0.06 | -0.3204 | 0.748665 | 97 | 0 | |
| HAGALIL HAELYON | 0.07 | 1.781663 | 0.074804 | 71 | 0 | |
| Hod Hasharon | 0.06 | -0.38796 | 0.698045 | 96 | 0 | |
| HAARAVA HATIKHONA | 0.11 | -0.45079 | 0.652139 | 4 | 0 | |
| HERZLIYYA | 0.07 | -0.16777 | 0.866764 | 93 | 0 | |
| Zemer | 0.09 | 0.905203 | 0.365358 | 106 | 0 | |
| HEVEL ELOT | 0.04 | -1.35158 | 0.176509 | 1 | 0 | |
| Hevel Modi'in | 0.06 | -1.68859 | 0.091298 | 93 | 0 | |
| Hadera | 0.12 | 1.330845 | 0.18324 | 97 | 0 | |
| Holon | 0.11 | -1.65292 | 0.098348 | 90 | 0 | |
| HOF ASHQELON | 0.11 | -0.49679 | 0.619335 | 55 | 0 | |
| Hof Hasharon | 0.05 | 0.093853 | 0.925226 | 89 | 0 | |
| TAYIBE | 0.12 | 0.307763 | 0.758263 | 89 | 0 | |
| TIRE | 0.17 | -0.65879 | 0.510033 | 91 | 0 | |
| Yavne | 0.11 | -1.79708 | 0.072323 | 87 | 0 | |
| KOKHAV YA'IR | 0.06 | -0.6254 | 0.53171 | 92 | 0 | |
| KAFAR BARA | 0.06 | -0.63896 | 0.522848 | 98 | 0 | |
| Kefar Yona | 0.08 | 0.481645 | 0.630058 | 90 | 0 | |
| KEFAR SAVA | 0.09 | -0.59908 | 0.549117 | 96 | 0 | |
| KAFAR QASEM | 0.15 | -0.70686 | 0.479653 | 97 | 0 | |
| MAJDAL SHAMS | 0.11 | -0.0399 | 0.968177 | 17 | 0 | |
| Menashe | 0.08 | 1.735066 | 0.082729 | 107 | 0 | |
| Mas'ade | 0.1 | 1.059822 | 0.289225 | 20 | 0 | |
| MIZPE RAMON | 0.04 | -0.81264 | 0.416427 | 3 | 0 | |
| NES ZIYYONA | 0.09 | -1.7949 | 0.07267 | 91 | 0 | |
| Netanya | 0.1 | 0.409589 | 0.682108 | 82 | 0 | |
| EMEQ HAMA'AYANOT | 0.09 | 0.691263 | 0.4894 | 65 | 0 | |
| PARDES HANNA-KARKUR | 0.07 | 1.640028 | 0.100999 | 105 | 0 | |
| PARDESIYYA | 0.15 | 0.35592 | 0.721901 | 86 | 0 | |
| PETAH TIQWA | 0.09 | -0.87688 | 0.38055 | 94 | 0 | |
| QADIMA-ZORAN | 0.09 | 0.396175 | 0.691976 | 85 | 0 | |
| QALANSAWE | 0.13 | 0.418996 | 0.675219 | 88 | 0 | |
| QAZRIN | 0.13 | 0.107497 | 0.914395 | 66 | 0 | |
| ROSH HAAYIN | 0.08 | -1.02133 | 0.307098 | 96 | 0 | |
| Ramat Gan | 0.11 | -1.58248 | 0.113539 | 93 | 0 | |
| Ramat Hasharon | 0.08 | -0.55682 | 0.577653 | 93 | 0 | |
| RA'ANNANA | 0.06 | -0.20609 | 0.836717 | 94 | 0 | |
| SEDEROT | 0.17 | -1.15195 | 0.249342 | 45 | 0 | |
| Sha'ar Hanegev | 0.13 | -1.63503 | 0.102044 | 45 | 0 | |
| Tel Aviv - Yafo | 0.1 | -1.43917 | 0.150102 | 89 | 0 | |
| Tel Mond | 0.04 | -0.40527 | 0.685278 | 89 | 0 | |
| ELAT | 0.12 | 0.771036 | 0.440685 | 1 | 0 | |
| QIRYAT ONO | 0.09 | -1.68842 | 0.09133 | 92 | 0 | |
| ORANIT | 0.05 | -0.63896 | 0.522848 | 98 | 0 | |
| ALFE MENASHE | 0.13 | -0.70248 | 0.482381 | 95 | 0 | |
| ELQANA | 0.03 | -0.81653 | 0.414194 | 99 | 0 | |
| ARI'EL | 0.07 | -1.22684 | 0.219882 | 91 | 0 | |
| GHAJAR | 0.05 | 0.514905 | 0.606619 | 31 | 0 | |
| IMMANU'EL | 0.06 | -0.87513 | 0.381504 | 93 | 0 | |
| QEDUMIM | 0.08 | -0.12623 | 0.899546 | 91 | 0 | |
| Gezer | 0.11 | -1.86559 | 0.062099 | 92 | -1 | |
| SEDOT NEGEV | 0.09 | -2.08886 | 0.036721 | 31 | -1 | |
| El'ad | 0.04 | -1.9008 | 0.057329 | 93 | -1 | |
| Gan Yavne | 0.09 | -2.05971 | 0.039426 | 81 | -1 | |
| Hevel Yavne | 0.07 | -2.0188 | 0.043508 | 83 | -1 | |
| YEHUD | 0.11 | -1.86541 | 0.062124 | 94 | -1 | |
| Savyon | 0.05 | -1.97153 | 0.048663 | 93 | -1 | |
| QIRYAT EQRON | 0.12 | -1.8304 | 0.06719 | 91 | -1 | |
| Rehovot | 0.09 | -1.86559 | 0.062099 | 92 | -1 | |
| Ramla | 0.12 | -2.07764 | 0.037743 | 93 | -1 | |
| EMEQ LOD | 0.07 | -1.9008 | 0.057329 | 93 | -1 | |
| Netivot | 0.08 | -1.92022 | 0.05483 | 37 | -1 | |
| QIRYAT MAL'AKHI | 0.11 | -2.13247 | 0.032968 | 81 | -1 | |
| Shoham | 0.07 | -1.86591 | 0.062055 | 91 | -1 | |
| BET ARYE | 0.07 | -1.97242 | 0.048562 | 91 | -1 | |
| MATTE YEHUDA | 0.07 | -2.59083 | 0.009575 | 85 | -2 | |
| ABU GHOSH | 0.06 | -2.61904 | 0.008818 | 78 | -2 | |
| BE'ER TUVEYA | 0.1 | -2.21164 | 0.026992 | 79 | -2 | |
| Be'er Sheva | 0.13 | -2.44624 | 0.014436 | 31 | -2 | |
| BET SHEMESH | 0.05 | -2.69879 | 0.006959 | 85 | -2 | |
| BENE AYISH | 0.15 | -2.19968 | 0.027829 | 83 | -2 | |
| BENE SHIM'ON | 0.09 | -2.58527 | 0.00973 | 39 | -2 | |
| BRENNER | 0.1 | -2.36612 | 0.017976 | 89 | -2 | |
| Gedera | 0.09 | -2.19496 | 0.028167 | 85 | -2 | |
| GEDEROT | 0.06 | -2.19496 | 0.028167 | 85 | -2 | |
| YO'AV | 0.05 | -2.39464 | 0.016637 | 79 | -2 | |
| JERUSALEM | 0.06 | -2.70575 | 0.006815 | 70 | -2 | |
| Lehavim | 0.1 | -2.88414 | 0.003925 | 42 | -2 | |
| Lod | 0.1 | -2.18544 | 0.028857 | 91 | -2 | |
| LAQYE | 0.02 | -2.96386 | 0.003038 | 37 | -2 | |
| MEVASSERET ZIYYON | 0.1 | -2.85124 | 0.004355 | 76 | -2 | |
| MODI'IN-MAKKABBIM-RE'UT | 0.07 | -2.29328 | 0.021832 | 90 | -2 | |
| Mazkeret Batya | 0.1 | -2.33227 | 0.019686 | 88 | -2 | |
| Merhavim | 0.07 | -2.29683 | 0.021629 | 29 | -2 | |
| NAHAL SOREQ | 0.05 | -2.26081 | 0.023771 | 88 | -2 | |
| Omer | 0.07 | -2.44624 | 0.014436 | 31 | -2 | |
| RISHON LEZIYYON | 0.1 | -2.22213 | 0.026274 | 90 | -2 | |
| Rahat | 0.05 | -2.75788 | 0.005818 | 46 | -2 | |
| SEGEV-SHALOM | 0.02 | -2.59342 | 0.009503 | 28 | -2 | |
| Shafir | 0.07 | -2.34331 | 0.019113 | 74 | -2 | |
| Tel Sheva | 0.05 | -2.63101 | 0.008513 | 30 | -2 | |
| BE'ER YA'AQOV | 0.09 | -2.18544 | 0.028857 | 91 | -2 | |
| QIRYAT GAT | 0.11 | -2.36555 | 0.018003 | 70 | -2 | |
| QIRYAT YE'ARIM | 0.05 | -2.54104 | 0.011052 | 79 | -2 | |
| MODI'IN ILLIT | 0.02 | -2.54232 | 0.011012 | 90 | -2 | |
| BET EL | 0.07 | -2.49257 | 0.012682 | 82 | -2 | |
| GIV'AT ZE'EV | 0.05 | -2.68267 | 0.007304 | 80 | -2 | |
| HAR ADAR | 0.06 | -2.68267 | 0.007304 | 80 | -2 | |
| Dimona | 0.11 | -3.14984 | 0.001634 | 20 | -3 | |
| AL-KASUM | 0.02 | -3.36521 | 0.000765 | 31 | -3 | |
| Hura | 0.02 | -3.34941 | 0.00081 | 34 | -3 | |
| KUSEIFE | 0.03 | -4.00803 | 6.12E-05 | 24 | -3 | |
| LAKHISH | 0.06 | -3.27471 | 0.001058 | 63 | -3 | |
| METAR | 0.13 | -3.217 | 0.001295 | 35 | -3 | |
| Neve Midbar | 0.01 | -3.15729 | 0.001592 | 25 | -3 | |
| Arad | 0.1 | -4.2374 | 2.26E-05 | 21 | -3 | |
| AR'ARA-BANEGEV | 0.02 | -3.68087 | 0.000232 | 22 | -3 | |
| Yeroham | 0.07 | -3.38961 | 0.0007 | 21 | -3 | |
| EFRAT | 0.04 | -3.88802 | 0.000101 | 59 | -3 | |
| BETAR ILLIT | 0.03 | -3.59043 | 0.00033 | 76 | -3 | |
| MA'ALE ADUMMIM | 0.1 | -3.62108 | 0.000293 | 40 | -3 | |
| QIRYAT ARBA | 0.04 | -3.599 | 0.000319 | 49 | -3 | |

|  |  |  |  |  |  |
| --- | --- | --- | --- | --- | --- |
|  |  |  |  |  |  |
|  |  |  |  |  |  |
|  |  |  |  |  |  |
|  |  |  |  |  |  |
|  |  |  |  |  |  |
|  |  |  |  |  |  |
|  |  |  |  |  |  |
|  |  |  |  |  |  |
|  |  |  |  |  |  |
|  |  |  |  |  |  |
|  |  |  |  |  |  |
|  |  |  |  |  |  |
|  |  |  |  |  |  |
|  |  |  |  |  |  |
|  |  |  |  |  |  |
|  |  |  |  |  |  |
|  |  |  |  |  |  |
|  |  |  |  |  |  |
|  |  |  |  |  |  |
|  |  |  |  |  |  |
|  |  |  |  |  |  |
|  |  |  |  |  |  |
|  |  |  |  |  |  |
|  |  |  |  |  |  |
|  |  |  |  |  |  |
|  |  |  |  |  |  |
|  |  |  |  |  |  |
|  |  |  |  |  |  |
|  |  |  |  |  |  |
|  |  |  |  |  |  |
|  |  |  |  |  |  |
|  |  |  |  |  |  |
|  |  |  |  |  |  |
|  |  |  |  |  |  |
|  |  |  |  |  |  |
|  |  |  |  |  |  |
|  |  |  |  |  |  |
|  |  |  |  |  |  |
|  |  |  |  |  |  |
|  |  |  |  |  |  |
|  |  |  |  |  |  |
|  |  |  |  |  |  |
|  |  |  |  |  |  |
|  |  |  |  |  |  |
|  |  |  |  |  |  |
|  |  |  |  |  |  |
|  |  |  |  |  |  |
|  |  |  |  |  |  |
|  |  |  |  |  |  |
|  |  |  |  |  |  |
|  |  |  |  |  |  |
|  |  |  |  |  |  |
|  |  |  |  |  |  |
|  |  |  |  |  |  |
|  |  |  |  |  |  |
|  |  |  |  |  |  |
|  |  |  |  |  |  |
|  |  |  |  |  |  |
|  |  |  |  |  |  |
|  |  |  |  |  |  |
|  |  |  |  |  |  |
|  |  |  |  |  |  |
|  |  |  |  |  |  |
|  |  |  |  |  |  |
|  |  |  |  |  |  |
|  |  |  |  |  |  |
|  |  |  |  |  |  |
|  |  |  |  |  |  |
|  |  |  |  |  |  |
|  |  |  |  |  |  |
|  |  |  |  |  |  |
|  |  |  |  |  |  |
|  |  |  |  |  |  |
|  |  |  |  |  |  |
|  |  |  |  |  |  |
|  |  |  |  |  |  |
|  |  |  |  |  |  |
|  |  |  |  |  |  |
|  |  |  |  |  |  |
|  |  |  |  |  |  |
|  |  |  |  |  |  |
|  |  |  |  |  |  |
|  |  |  |  |  |  |
|  |  |  |  |  |  |
|  |  |  |  |  |  |
|  |  |  |  |  |  |
|  |  |  |  |  |  |
|  |  |  |  |  |  |
|  |  |  |  |  |  |
|  |  |  |  |  |  |
|  |  |  |  |  |  |
|  |  |  |  |  |  |
|  |  |  |  |  |  |
|  |  |  |  |  |  |
|  |  |  |  |  |  |
|  |  |  |  |  |  |
|  |  |  |  |  |  |
|  |  |  |  |  |  |
|  |  |  |  |  |  |
|  |  |  |  |  |  |
|  |  |  |  |  |  |
|  |  |  |  |  |  |
|  |  |  |  |  |  |
|  |  |  |  |  |  |
|  |  |  |  |  |  |
|  |  |  |  |  |  |
|  |  |  |  |  |  |
|  |  |  |  |  |  |
|  |  |  |  |  |  |
|  |  |  |  |  |  |
|  |  |  |  |  |  |
|  |  |  |  |  |  |
|  |  |  |  |  |  |
|  |  |  |  |  |  |
|  |  |  |  |  |  |
|  |  |  |  |  |  |
|  |  |  |  |  |  |
|  |  |  |  |  |  |
|  |  |  |  |  |  |
|  |  |  |  |  |  |
|  |  |  |  |  |  |
|  |  |  |  |  |  |
|  |  |  |  |  |  |
|  |  |  |  |  |  |
|  |  |  |  |  |  |
|  |  |  |  |  |  |
|  |  |  |  |  |  |
|  |  |  |  |  |  |
|  |  |  |  |  |  |
|  |  |  |  |  |  |
|  |  |  |  |  |  |
|  |  |  |  |  |  |
|  |  |  |  |  |  |
|  |  |  |  |  |  |
|  |  |  |  |  |  |
|  |  |  |  |  |  |
|  |  |  |  |  |  |
|  |  |  |  |  |  |
|  |  |  |  |  |  |
|  |  |  |  |  |  |
|  |  |  |  |  |  |
|  |  |  |  |  |  |
|  |  |  |  |  |  |
|  |  |  |  |  |  |
|  |  |  |  |  |  |
|  |  |  |  |  |  |
|  |  |  |  |  |  |
|  |  |  |  |  |  |
|  |  |  |  |  |  |
|  |  |  |  |  |  |
|  |  |  |  |  |  |
|  |  |  |  |  |  |
|  |  |  |  |  |  |
|  |  |  |  |  |  |
|  |  |  |  |  |  |
|  |  |  |  |  |  |
|  |  |  |  |  |  |
|  |  |  |  |  |  |
|  |  |  |  |  |  |
|  |  |  |  |  |  |
|  |  |  |  |  |  |
|  |  |  |  |  |  |
|  |  |  |  |  |  |
|  |  |  |  |  |  |
|  |  |  |  |  |  |
|  |  |  |  |  |  |
|  |  |  |  |  |  |
|  |  |  |  |  |  |
|  |  |  |  |  |  |
|  |  |  |  |  |  |
|  |  |  |  |  |  |
|  |  |  |  |  |  |
|  |  |  |  |  |  |
|  |  |  |  |  |  |
|  |  |  |  |  |  |
|  |  |  |  |  |  |
|  |  |  |  |  |  |
|  |  |  |  |  |  |
|  |  |  |  |  |  |
|  |  |  |  |  |  |
|  |  |  |  |  |  |
|  |  |  |  |  |  |
|  |  |  |  |  |  |
|  |  |  |  |  |  |
|  |  |  |  |  |  |
|  |  |  |  |  |  |
|  |  |  |  |  |  |
|  |  |  |  |  |  |
|  |  |  |  |  |  |
|  |  |  |  |  |  |
|  |  |  |  |  |  |
|  |  |  |  |  |  |
|  |  |  |  |  |  |
|  |  |  |  |  |  |
|  |  |  |  |  |  |
|  |  |  |  |  |  |
|  |  |  |  |  |  |
|  |  |  |  |  |  |
|  |  |  |  |  |  |
|  |  |  |  |  |  |
|  |  |  |  |  |  |
|  |  |  |  |  |  |
|  |  |  |  |  |  |
|  |  |  |  |  |  |
|  |  |  |  |  |  |
|  |  |  |  |  |  |
|  |  |  |  |  |  |
|  |  |  |  |  |  |
|  |  |  |  |  |  |
|  |  |  |  |  |  |
|  |  |  |  |  |  |
|  |  |  |  |  |  |
|  |  |  |  |  |  |
|  |  |  |  |  |  |
|  |  |  |  |  |  |
|  |  |  |  |  |  |
|  |  |  |  |  |  |
|  |  |  |  |  |  |
|  |  |  |  |  |  |
|  |  |  |  |  |  |
|  |  |  |  |  |  |
|  |  |  |  |  |  |
|  |  |  |  |  |  |
|  |  |  |  |  |  |
|  |  |  |  |  |  |
|  |  |  |  |  |  |
|  |  |  |  |  |  |
|  |  |  |  |  |  |
|  |  |  |  |  |  |
|  |  |  |  |  |  |
|  |  |  |  |  |  |
|  |  |  |  |  |  |
|  |  |  |  |  |  |
|  |  |  |  |  |  |
