# Supplementary material for: Spatial patterns of childhood obesity clusters linked to socioeconomic inequalities
Source: Front Public Health. 2025 Aug 19;13:1497090. doi: 10.3389/fpubh.2025.1497090 (PMC12404039; doi:10.3389/fpubh.2025.1497090)
Supplement: Supplementary file 3 [file Table_2.docx]

**Table S2.** **Hot spot analysis results for 2018.**

| **Ob_Per** | **GiZScore** | **GiPValue** | **NNeighbors** | **Gi_Bin** | **Locality Name** |
| --- | --- | --- | --- | --- | --- |
| 0.08 | 5.637362 | 1.73E-08 | 109 | 3 | Emek Yizrael |
| 0.06 | 5.472323 | 4.44E-08 | 107 | 3 | Zevulun |
| 0.06 | 4.524204 | 6.06E-06 | 114 | 3 | Megido |
| 0.13 | 4.132972 | 3.58E-05 | 113 | 3 | Alona |
| 0.11 | 4.465963 | 7.97E-06 | 98 | 3 | Abu Snan |
| 0.12 | 4.374605 | 1.22E-05 | 119 | 3 | Um al Fahem |
| 0.13 | 5.603257 | 2.1E-08 | 109 | 3 | Iksal |
| 0.08 | 5.199123 | 2E-07 | 107 | 3 | al Batuf |
| 0.12 | 5.540623 | 3.01E-08 | 107 | 3 | I'eblin |
| 0.24 | 5.418187 | 6.02E-08 | 108 | 3 | Bustan al Marj |
| 0.06 | 4.473643 | 7.69E-06 | 104 | 3 | Bu'eyne - Nujidat |
| 0.04 | 5.637362 | 1.73E-08 | 109 | 3 | Bir al Maksur |
| 0.06 | 3.867292 | 0.00011 | 97 | 3 | Beit Jan |
| 0.08 | 5.452313 | 4.97E-08 | 108 | 3 | Basmat Tivon |
| 0.09 | 4.330899 | 1.49E-05 | 101 | 3 | Ba'ana |
| 0.11 | 4.937172 | 7.93E-07 | 100 | 3 | Jdeyde - Ma'ker |
| 0.17 | 4.490102 | 7.12E-06 | 100 | 3 | Julis |
| 0.22 | 3.204767 | 0.001352 | 91 | 3 | Jiser al Zarka |
| 0.09 | 3.274562 | 0.001058 | 91 | 3 | Jish (Gush Halav) |
| 0.11 | 5.428906 | 5.67E-08 | 111 | 3 | Daliat al carmel |
| 0.12 | 5.432733 | 5.55E-08 | 109 | 3 | Daburiya |
| 0.08 | 4.386932 | 1.15E-05 | 100 | 3 | Dir al Asad |
| 0.13 | 4.494373 | 6.98E-06 | 103 | 3 | Dir Hana |
| 0.13 | 4.824259 | 1.41E-06 | 102 | 3 | Hagilbo'a |
| 0.06 | 4.378252 | 1.2E-05 | 102 | 3 | Hagalil Hatahton |
| 0.07 | 4.307272 | 1.65E-05 | 109 | 3 | Zih'ron Ya'akov |
| 0.1 | 5.588818 | 2.29E-08 | 108 | 3 | Zarzir |
| 0.07 | 4.550272 | 5.36E-06 | 107 | 3 | Hof Hacarmel |
| 0.07 | 3.762073 | 0.000169 | 94 | 3 | Hurfeish |
| 0.08 | 5.407498 | 6.39E-08 | 102 | 3 | Haifa |
| 0.05 | 3.076178 | 0.002097 | 89 | 3 | Hatsor Haglilit |
| 0.1 | 3.993438 | 6.51E-05 | 96 | 3 | Tverya (Tiberias) |
| 0.07 | 3.369908 | 0.000752 | 87 | 3 | Tuba - Zangariya |
| 0.07 | 4.487492 | 7.21E-06 | 105 | 3 | Tur'an |
| 0.18 | 5.603894 | 2.1E-08 | 98 | 3 | Tirat Karmel |
| 0.11 | 5.321886 | 1.03E-07 | 106 | 3 | Tamra |
| 0.1 | 4.143559 | 3.42E-05 | 97 | 3 | Yanuh - Jat |
| 0.06 | 4.005425 | 6.19E-05 | 97 | 3 | Yavne'el |
| 0.1 | 5.588818 | 2.29E-08 | 108 | 3 | Yafi'a |
| 0.11 | 5.051715 | 4.38E-07 | 115 | 3 | Yokne'am Ilit |
| 0.07 | 4.340137 | 1.42E-05 | 99 | 3 | Yirka |
| 0.11 | 5.171601 | 2.32E-07 | 105 | 3 | Kabul |
| 0.15 | 5.199123 | 2E-07 | 107 | 3 | Kawkab Abu al Hija |
| 0.04 | 4.028025 | 5.62E-05 | 96 | 3 | Kisrah - Smi'a |
| 0.11 | 5.588818 | 2.29E-08 | 108 | 3 | Ka'abiya - Tabash - Hajajre |
| 0.06 | 3.912439 | 9.14E-05 | 95 | 3 | Kefar Veradim |
| 0.12 | 4.490102 | 7.12E-06 | 100 | 3 | Kefar Yasif |
| 0.09 | 4.50788 | 6.55E-06 | 104 | 3 | Kefar Kama |
| 0.08 | 5.349935 | 8.8E-08 | 108 | 3 | Kefar Kana |
| 0.12 | 5.349935 | 8.8E-08 | 108 | 3 | Kefar Manda |
| 0.11 | 3.45465 | 0.000551 | 109 | 3 | Kefar Kara |
| 0.06 | 4.721022 | 2.35E-06 | 107 | 3 | Kefar Tavor |
| 0.09 | 4.460102 | 8.19E-06 | 103 | 3 | Karmiel |
| 0.12 | 3.031479 | 0.002434 | 78 | 3 | Mevo'ot Hahermon |
| 0.12 | 4.365247 | 1.27E-05 | 101 | 3 | Majd al Kerum |
| 0.12 | 5.637362 | 1.73E-08 | 109 | 3 | Migdal Ha'emek |
| 0.16 | 4.15494 | 3.25E-05 | 95 | 3 | Mazra'a |
| 0.09 | 4.166372 | 3.09E-05 | 96 | 3 | Ma'te Asher |
| 0.07 | 3.889593 | 0.0001 | 93 | 3 | Ma'ale Yosef |
| 0.08 | 3.715771 | 0.000203 | 93 | 3 | Ma'alot - Tarshiha |
| 0.05 | 3.588562 | 0.000333 | 94 | 3 | Merom Hagalil |
| 0.04 | 4.494373 | 6.98E-06 | 103 | 3 | Misgav |
| 0.06 | 5.432733 | 5.55E-08 | 109 | 3 | Mishad |
| 0.1 | 5.432733 | 5.55E-08 | 109 | 3 | Nof Hagalil |
| 0.08 | 4.443442 | 8.85E-06 | 99 | 3 | Nahef |
| 0.1 | 5.54992 | 2.86E-08 | 110 | 3 | Natsrat (Nazareth) |
| 0.18 | 5.500754 | 3.78E-08 | 104 | 3 | Nesher |
| 0.09 | 4.121135 | 3.77E-05 | 98 | 3 | Sajur |
| 0.11 | 4.494373 | 6.98E-06 | 103 | 3 | Sah'nin |
| 0.1 | 5.444622 | 5.19E-08 | 112 | 3 | Osfiya |
| 0.08 | 4.494373 | 6.98E-06 | 103 | 3 | Ilabun |
| 0.09 | 5.54992 | 2.86E-08 | 110 | 3 | Ilut |
| 0.12 | 5.030108 | 4.9E-07 | 102 | 3 | Ako (Acre) |
| 0.12 | 3.739255 | 0.000185 | 92 | 3 | Emek Hayarden |
| 0.12 | 4.494373 | 6.98E-06 | 103 | 3 | Ara'be |
| 0.12 | 4.316465 | 1.59E-05 | 105 | 3 | Furaydis |
| 0.16 | 3.762073 | 0.000169 | 94 | 3 | Fasuta |
| 0.12 | 3.936359 | 8.27E-05 | 97 | 3 | Pki'in (Buke'a) |
| 0.07 | 3.55386 | 0.00038 | 94 | 3 | Tsefat |
| 0.09 | 5.466517 | 4.59E-08 | 104 | 3 | Kiryat Biyalik |
| 0.04 | 5.432733 | 5.55E-08 | 109 | 3 | Kiryat Tiv'on |
| 0.13 | 5.316884 | 1.06E-07 | 103 | 3 | Kityat Yam |
| 0.12 | 4.180592 | 2.91E-05 | 100 | 3 | Ra'me |
| 0.1 | 5.515834 | 3.47E-08 | 110 | 3 | Reyne |
| 0.05 | 5.506473 | 3.66E-08 | 107 | 3 | Rehasim |
| 0.04 | 5.37949 | 7.47E-08 | 110 | 3 | Ramat Yishay |
| 0.07 | 5.432733 | 5.55E-08 | 109 | 3 | Shibli - Um al Ranem |
| 0.1 | 3.313803 | 0.00092 | 85 | 3 | Shelomi |
| 0.13 | 4.952961 | 7.31E-07 | 104 | 3 | Sha'ab |
| 0.09 | 5.622944 | 1.88E-08 | 108 | 3 | Shfar'am |
| 0.13 | 3.8441 | 0.000121 | 116 | 3 | Basma |
| 0.07 | 4.399595 | 1.08E-05 | 101 | 3 | Mughar |
| 0.1 | 4.340575 | 1.42E-05 | 119 | 3 | Ma'ale I'ron |
| 0.12 | 3.787404 | 0.000152 | 90 | 3 | Nahariya |
| 0.12 | 5.10406 | 3.32E-07 | 112 | 3 | Afula |
| 0.07 | 3.727489 | 0.000193 | 109 | 3 | Ar'ara |
| 0.1 | 5.582066 | 2.38E-08 | 105 | 3 | Kiryat Ata |
| 0.15 | 5.351156 | 8.74E-08 | 103 | 3 | Kiryat Motskin |
| 0.16 | 2.244225 | 0.024818 | 97 | 2 | Or Akiva |
| 0.13 | 2.312705 | 0.020739 | 108 | 2 | Baka al Rarbiya |
| 0.13 | 2.518899 | 0.011772 | 70 | 2 | Beit She'an |
| 0.08 | 2.19392 | 0.028241 | 107 | 2 | Menashe |
| 0.08 | 2.589148 | 0.009621 | 65 | 2 | Emek Hama'ayanot |
| 0.08 | 2.161521 | 0.030655 | 105 | 2 | Pardes Hana - Karkur |
| 0.13 | 2.024699 | 0.042898 | 105 | 1 | jat |
| 0.07 | 1.973132 | 0.048481 | 71 | 1 | Hagalil Ha'elyon |
| 0.11 | 2.002491 | 0.045232 | 97 | 1 | Hadera |
| 0.09 | 1.916462 | 0.055306 | 66 | 1 | Katserin |
| 0.06 | 0.812387 | 0.41657 | 94 | 0 | Emek Hefer |
| 0.05 | 0.517979 | 0.604473 | 55 | 0 | Golan |
| 0.04 | -0.06333 | 0.9495 | 86 | 0 | Lev Hasharon |
| 0.07 | -0.25895 | 0.79567 | 85 | 0 | Even Yehuda |
| 0.12 | -0.45283 | 0.650673 | 53 | 0 | Ashkelon |
| 0.07 | 0.032882 | 0.973768 | 22 | 0 | Buk'ata |
| 0.1 | -1.00191 | 0.316387 | 97 | 0 | Jaljulya |
| 0.05 | -1.00191 | 0.316387 | 97 | 0 | Drom Hasharon |
| 0.07 | -1.05623 | 0.290865 | 96 | 0 | Hod Hasharon |
| 0.09 | -0.77402 | 0.438917 | 4 | 0 | Ha'arava Hatihona |
| 0.07 | -0.83836 | 0.401831 | 93 | 0 | Hertseliya |
| 0.11 | 1.323242 | 0.185755 | 106 | 0 | Zemer |
| 0.04 | -1.16691 | 0.243246 | 1 | 0 | Hevel Eielot |
| 0.11 | -0.92639 | 0.354241 | 55 | 0 | Hof Ashkelon |
| 0.07 | -0.4632 | 0.64322 | 89 | 0 | Hof Hasharon |
| 0.13 | -0.28798 | 0.773359 | 89 | 0 | Taybe |
| 0.15 | -0.91316 | 0.361161 | 91 | 0 | Tira |
| 0.01 | -0.96272 | 0.335687 | 92 | 0 | Kohav Yair |
| 0.23 | -1.25818 | 0.208327 | 98 | 0 | Kefar Bara |
| 0.08 | -0.12912 | 0.897266 | 90 | 0 | Kefar Yona |
| 0.06 | -1.1254 | 0.26042 | 96 | 0 | Kefar Saba |
| 0.11 | -1.31271 | 0.189281 | 97 | 0 | Kefar Kasem |
| 0.05 | -0.26638 | 0.78995 | 17 | 0 | Majdel Shams |
| 0.11 | 0.092056 | 0.926654 | 20 | 0 | Mas'ade |
| 0.05 | -0.66887 | 0.503578 | 3 | 0 | Mitspe Ramon |
| 0.09 | -0.10332 | 0.917712 | 82 | 0 | Netanya |
| 0.01 | -0.45148 | 0.651642 | 86 | 0 | Pardesiya |
| 0.08 | -1.68617 | 0.091763 | 94 | 0 | Petah Tikva |
| 0.05 | -0.18821 | 0.850714 | 85 | 0 | Kadima - Tsoran |
| 0.14 | -0.27196 | 0.785651 | 88 | 0 | Kalanswa |
| 0.12 | 1.526459 | 0.126895 | 48 | 0 | Kiryat Shemona |
| 0.05 | -1.22076 | 0.222176 | 93 | 0 | Ramat Hasharon |
| 0.04 | -0.95742 | 0.338353 | 94 | 0 | Ra'anana |
| 0.05 | -0.81364 | 0.415854 | 89 | 0 | Tel Mond |
| 0.1 | 0.397887 | 0.690714 | 1 | 0 | Eilat |
| 0.04 | -1.25818 | 0.208327 | 98 | 0 | ORANIT |
| 0.05 | -1.18008 | 0.237967 | 95 | 0 | ALFE MENASHE |
| 0.05 | -1.37608 | 0.168798 | 99 | 0 | ELQANA |
| 0.1 | -0.18593 | 0.852496 | 31 | 0 | GHAJAR |
| 0.03 | -1.186 | 0.235623 | 93 | 0 | IMMANU'EL |
| 0.02 | -0.00582 | 0.995359 | 91 | 0 | QEDUMIM |
| 0.09 | -1.95314 | 0.050803 | 75 | -1 | Ashdod |
| 0.04 | -1.79863 | 0.072077 | 92 | -1 | Bnei Berak |
| 0.06 | -1.74796 | 0.080471 | 96 | -1 | Rosh Ha'ayin |
| 0.06 | -2.02988 | 0.042369 | 91 | -1 | ARIEL |
| 0.07 | -2.41998 | 0.015521 | 34 | -2 | Sdot Negev |
| 0.1 | -2.6461 | 0.008143 | 93 | -2 | Or Yehuda |
| 0.09 | -2.51845 | 0.011787 | 91 | -2 | Azor |
| 0.04 | -2.57657 | 0.009979 | 93 | -2 | El'ad |
| 0.08 | -2.31911 | 0.020389 | 21 | -2 | Eshkol |
| 0.11 | -2.57132 | 0.010131 | 79 | -2 | Be'er Tuvya |
| 0.12 | -2.63454 | 0.008425 | 92 | -2 | Beit Dagan |
| 0.12 | -2.68185 | 0.007322 | 90 | -2 | Bat Yam |
| 0.06 | -2.42556 | 0.015285 | 92 | -2 | Giv'at Shemuel |
| 0.06 | -2.39073 | 0.016815 | 92 | -2 | Giv'atayim |
| 0.08 | -2.51845 | 0.011787 | 91 | -2 | Ganei Tikva |
| 0.08 | -2.60907 | 0.009079 | 83 | -2 | Hevel Yavne |
| 0.17 | -2.57657 | 0.009979 | 93 | -2 | Hevel Modi'in |
| 0.1 | -2.68185 | 0.007322 | 90 | -2 | Holon |
| 0.09 | -2.69253 | 0.007091 | 94 | -2 | Yehud - Monoson |
| 0.06 | -2.36799 | 0.017885 | 93 | -2 | Ramat Gan |
| 0.11 | -2.1301 | 0.033163 | 45 | -2 | Sderot |
| 0.08 | -2.47544 | 0.013307 | 45 | -2 | Sha'ar Hanegev |
| 0.06 | -2.25041 | 0.024423 | 89 | -2 | Tel Aviv - Yafo |
| 0.06 | -2.49522 | 0.012588 | 92 | -2 | Kiryat Ono |
| 0.01 | -2.69293 | 0.007083 | 91 | -2 | BET ARYE |
| 0.1 | -2.61993 | 0.008795 | 40 | -2 | MA'ALE ADUMMIM |
| 0.07 | -3.68066 | 0.000233 | 20 | -3 | Dimona |
| 0.07 | -3.47797 | 0.000505 | 85 | -3 | Ma'te Yehuda |
| 0.07 | -3.26147 | 0.001108 | 92 | -3 | Gezer |
| 0.07 | -3.24801 | 0.001162 | 78 | -3 | Abu Gosh (Abu Rosh) |
| 0.09 | -3.19691 | 0.001389 | 31 | -3 | Ofakim |
| 0.03 | -4.4013 | 1.08E-05 | 31 | -3 | al Kasum |
| 0.11 | -3.54819 | 0.000388 | 31 | -3 | Be'er Sheva |
| 0.06 | -3.4426 | 0.000576 | 85 | -3 | Beit Shemesh |
| 0.1 | -3.14247 | 0.001675 | 83 | -3 | Bnei Ayish |
| 0.05 | -3.67478 | 0.000238 | 39 | -3 | Bnei Shim'on |
| 0.07 | -3.09145 | 0.001992 | 89 | -3 | Brener |
| 0.11 | -3.15961 | 0.00158 | 85 | -3 | Gedera |
| 0.07 | -3.15961 | 0.00158 | 85 | -3 | Gderot |
| 0.08 | -2.91168 | 0.003595 | 81 | -3 | Gan Yavne |
| 0.06 | -2.88119 | 0.003962 | 89 | -3 | Gan Ra've |
| 0.03 | -4.20619 | 2.6E-05 | 34 | -3 | Hura |
| 0.11 | -3.07205 | 0.002126 | 87 | -3 | Yavne |
| 0.06 | -3.39875 | 0.000677 | 79 | -3 | Yoav |
| 0.06 | -2.79422 | 0.005202 | 70 | -3 | Yerushalayim (Jerusalem) |
| 0.03 | -4.4515 | 8.53E-06 | 24 | -3 | Kseyfe |
| 0 | -3.67608 | 0.000237 | 42 | -3 | Lehavim |
| 0.09 | -3.11171 | 0.00186 | 91 | -3 | Lod |
| 0.05 | -3.90773 | 9.32E-05 | 63 | -3 | Lahish |
| 0.06 | -3.80261 | 0.000143 | 37 | -3 | Lakiye |
| 0.07 | -3.45309 | 0.000554 | 76 | -3 | Mevaseret Tsiyon |
| 0.05 | -3.34626 | 0.000819 | 90 | -3 | Modi'in - Makabim - Re'ut |
| 0.12 | -3.11676 | 0.001828 | 88 | -3 | Mazkeret Batya |
| 0.08 | -4.1787 | 2.93E-05 | 35 | -3 | Meitar |
| 0.04 | -3.39506 | 0.000686 | 29 | -3 | Merhavim |
| 0.02 | -4.01259 | 6.01E-05 | 25 | -3 | Neve Midbar |
| 0.05 | -3.39773 | 0.000679 | 88 | -3 | Nahal Sorek |
| 0.06 | -3.1815 | 0.001465 | 91 | -3 | Nes Tsiyona |
| 0.12 | -2.81992 | 0.004804 | 93 | -3 | Savyon |
| 0.06 | -3.54819 | 0.000388 | 31 | -3 | Omer |
| 0.1 | -4.52146 | 6.14E-06 | 21 | -3 | Arad |
| 0.02 | -4.10582 | 4.03E-05 | 22 | -3 | Ar'ara Banegev |
| 0.15 | -3.2513 | 0.001149 | 91 | -3 | Kiryat Ekron |
| 0.09 | -3.17141 | 0.001517 | 90 | -3 | Rishon Letsiyon |
| 0.05 | -3.45978 | 0.000541 | 46 | -3 | Rahat |
| 0.07 | -3.26147 | 0.001108 | 92 | -3 | Rehovot |
| 0.1 | -3.23709 | 0.001208 | 93 | -3 | Ramla |
| 0.04 | -3.89364 | 9.88E-05 | 28 | -3 | Segev Shalom |
| 0.07 | -2.85469 | 0.004308 | 93 | -3 | Sdot Dan |
| 0.04 | -2.96718 | 0.003005 | 74 | -3 | Shafir |
| 0.05 | -3.7774 | 0.000158 | 30 | -3 | Tel Sheva |
| 0.1 | -3.11171 | 0.00186 | 91 | -3 | Be'er Ya'akov |
| 0.07 | -3.80719 | 0.000141 | 21 | -3 | Yeroham |
| 0.09 | -2.82341 | 0.004752 | 37 | -3 | Netivot |
| 0.09 | -2.75707 | 0.005832 | 70 | -3 | Kiryat Gat |
| 0.07 | -3.14692 | 0.00165 | 79 | -3 | Kiryat Ye'arim |
| 0.08 | -2.73288 | 0.006278 | 81 | -3 | Kiryat Mal'ahi |
| 0.06 | -2.79763 | 0.005148 | 91 | -3 | Shoham |
| 0.02 | -3.55607 | 0.000376 | 90 | -3 | MODI'IN ILLIT |
| 0.05 | -3.48632 | 0.00049 | 59 | -3 | EFERAT |
| 0.04 | -3.38385 | 0.000715 | 82 | -3 | BET EL |
| 0.03 | -3.3441 | 0.000825 | 76 | -3 | BETAR ILLIT |
| 0.06 | -3.19021 | 0.001422 | 80 | -3 | GIV'AT ZE'EV |
| 0 | -3.19021 | 0.001422 | 80 | -3 | HAR ADAR |
| 0.02 | -4.35863 | 1.31E-05 | 49 | -3 | QIRYAT ARBA |
