# Supplementary material for: Spatial patterns of childhood obesity clusters linked to socioeconomic inequalities
Source: Front Public Health. 2025 Aug 19;13:1497090. doi: 10.3389/fpubh.2025.1497090 (PMC12404039; doi:10.3389/fpubh.2025.1497090)
Supplement: Supplementary file 4 [file Table_3.docx]

**Table S3. Correlation matrix of the socioeconomic and demographic parameters.**

| A | Obesity percentage |
| --- | --- |
| B | Average number of days abourd |
| C | Average vehicle licence fee |
| D | Rate of motorization |
| E | Average monthly income per capita |
| F | Percent of recipients of income support and income supplement to old age pension |
| G | Percent of wege and income earners below the minimum wedge |
| H | Percent of wage and income earners above twice the average wage |
| I | Percent of women aged 25-54 with no income from work |
| J | Percent of wage and income earners of aged 15 and over |
| K | Percent of academic degree holders of aged 25-54 |

|  | A | B | C | D | E | F | G | H | I | J | K |
| --- | --- | --- | --- | --- | --- | --- | --- | --- | --- | --- | --- |
| A | 1 | -0.10958 | -0.18852 | 0.123305 | 0.018181 | -0.24008 | 0.093196 | -0.14862 | 0.155771 | 0.177659 | -0.14835 |
| B | -0.1095782 | 1 | 0.710715 | 0.776353 | 0.935877 | 0.547953 | 0.802888 | 0.917292 | 0.627549 | 0.612805 | 0.859456 |
| C | -0.1885191 | 0.710715 | 1 | 0.626257 | 0.662085 | 0.4084 | 0.503287 | 0.680289 | 0.180773 | 0.257282 | 0.577687 |
| D | 0.12330485 | 0.776353 | 0.626257 | 1 | 0.869653 | 0.510153 | 0.775354 | 0.809412 | 0.491644 | 0.671706 | 0.75961 |
| E | 0.01818133 | 0.935877 | 0.662085 | 0.869653 | 1 | 0.544929 | 0.902253 | 0.955031 | 0.70177 | 0.743696 | 0.884423 |
| F | -0.240077 | 0.547953 | 0.4084 | 0.510153 | 0.544929 | 1 | 0.520944 | 0.636762 | 0.500038 | 0.563349 | 0.635778 |
| G | 0.09319563 | 0.802888 | 0.503287 | 0.775354 | 0.902253 | 0.520944 | 1 | 0.850081 | 0.72885 | 0.758583 | 0.802933 |
| H | -0.148616 | 0.917292 | 0.680289 | 0.809412 | 0.955031 | 0.636762 | 0.850081 | 1 | 0.621255 | 0.655076 | 0.940422 |
| I | 0.15577055 | 0.627549 | 0.180773 | 0.491644 | 0.70177 | 0.500038 | 0.72885 | 0.621255 | 1 | 0.872801 | 0.643331 |
| J | 0.17765943 | 0.612805 | 0.257282 | 0.671706 | 0.743696 | 0.563349 | 0.758583 | 0.655076 | 0.872801 | 1 | 0.664491 |
| K | -0.148347 | 0.859456 | 0.577687 | 0.75961 | 0.884423 | 0.635778 | 0.802933 | 0.940422 | 0.643331 | 0.664491 | 1 |

|  |  |  |  |  |  |  |  |  |  |  |  |
| --- | --- | --- | --- | --- | --- | --- | --- | --- | --- | --- | --- |
|  |  |  |  |  |  |  |  |  |  |  |  |
|  |  |  |  |  |  |  |  |  |  |  |  |
|  |  |  |  |  |  |  |  |  |  |  |  |
|  |  |  |  |  |  |  |  |  |  |  |  |
|  |  |  |  |  |  |  |  |  |  |  |  |
|  |  |  |  |  |  |  |  |  |  |  |  |
|  |  |  |  |  |  |  |  |  |  |  |  |
|  |  |  |  |  |  |  |  |  |  |  |  |
|  |  |  |  |  |  |  |  |  |  |  |  |
